# Association of fluvoxamine with mortality and symptom resolution among inpatients with COVID-19 in Uganda: a prospective interventional open-label cohort study

**DOI:** 10.1038/s41380-023-02004-3

**Published:** 2023-03-03

**Authors:** Bruce J. Kirenga, Levicatus Mugenyi, Marina Sánchez-Rico, Henry Kyobe, Winters Muttamba, Raymond Mugume, Eliya Mwesigwa, Ezra Kalimo, Vicky Nyombi, Ivan Segawa, Loryndah Olive Namakula, Rogers Sekibira, Wilberforce Kabweru, Rosemary Byanyima, Hellen Aanyu, Pauline Byakika-Kibwika, Henry G. Mwebesa, Nicolas Hoertel, William Bazeyo

**Affiliations:** 1 Department of Internal Medicine, Makerere University, Kampala, Uganda; 2 Makerere University Lung Institute, Kampala, Uganda; 3 Medical Research Council, Uganda Virus Research Institute and London School of Hygiene and Tropical Medicine, Entebbe Unit, Entebbe, Uganda; 4 Assistance Publique–Hôpitaux de Paris (AP-HP), DMU Psychiatrie et Addictologie, Hôpital Corentin-Celton, F-92130 Issy-les-Moulineaux, France; 5 Ministry of Health Uganda, Kampala, Uganda; 6 Division of Infection and Global Health, School of Medicine, University of St Andrews, St Andrews, UK; 7 Mulago National Referral Hospital, Kampala, Uganda; 8 Université Paris Cité, Paris, France; 9 INSERM U1266, Institut de Psychiatrie et Neuroscience de Paris, Paris, France; 10 Makerere University, Kampala, Uganda

**Keywords:** Diseases, Physiology, Prognostic markers

## Abstract

Prior research suggests that fluvoxamine, a selective serotonin reuptake inhibitor (SSRI) used for the treatment of obsessive-compulsive disorder and major depressive disorder, could be repurposed against COVID-19. We undertook a prospective interventional open-label cohort study to evaluate the efficacy and tolerability of fluvoxamine among inpatients with laboratory-confirmed COVID-19 in Uganda. The main outcome was all-cause mortality. Secondary outcomes were hospital discharge and complete symptom resolution. We included 316 patients, of whom 94 received fluvoxamine in addition to standard care [median age, 60 years (IQR = 37.0); women, 52.2%]. Fluvoxamine use was significantly associated with reduced mortality [AHR = 0.32; 95% CI = 0.19–0.53; *p* < 0.001, NNT = 4.46] and with increased complete symptom resolution [AOR = 2.56; 95% CI = 1.53–5.51; *p* < 0.001, NNT = 4.44]. Sensitivity analyses yielded similar results. These effects did not significantly differ by clinical characteristic, including vaccination status. Among the 161 survivors, fluvoxamine was not significantly associated with time to hospital discharge [AHR 0.81, 95% CI (0.54–1.23), *p* = 0.32]. There was a trend toward greater side effects with fluvoxamine (7.45% versus 3.15%; SMD = 0.21; *χ*^2^ = 3.46, *p* = 0.06), most of which were light or mild in severity and none of which were serious. One hundred mg of fluvoxamine prescribed twice daily for 10 days was well tolerated and significantly associated with reduced mortality and with increased complete symptom resolution, without a significant increase in time to hospital discharge, among inpatients with COVID-19. Large-scale randomized trials are urgently needed to confirm these findings, especially for low- and middle-income countries, where access to vaccines and approved treatments against COVID-19 is limited.

## Introduction

The SARS-CoV-2 pandemic has created a tremendous economic and health crisis worldwide [1–3] and has led to excess mortality, especially in low- and middle-income countries [4, 5]. Because a large portion of the world’s population is currently unvaccinated [6], effective treatments for COVID-19—especially those that can be administered orally, have good tolerability, low rates of medical contraindications [7, 8], and wide availability at low cost—are urgently needed to reduce COVID-19-related mortality and morbidity [9–11]. This is particularly important in low- and middle-income countries, where access to vaccines and approved treatments against COVID-19 is limited [9–11].

Prior research suggests that fluvoxamine, a selective serotonin reuptake inhibitor (SSRI) used for the treatment of obsessive-compulsive disorder and major depressive disorder [12], could be repurposed against COVID-19 [9–11, 13–16]. In the ambulatory setting, three studies, including two randomized, placebo-controlled trials (RCTs) and one nonrandomized open-label clinical study, found a significant association between the short-term use (10–15 days) of fluvoxamine prescribed at a daily dose between 100 and 300 mg and taken within 7 days of symptom onset and a reduced risk of clinical deterioration [17–19]. A prospective cohort study of patients admitted to intensive care units (ICUs) for COVID-19 also reported a significant association between the 15-day use of fluvoxamine prescribed at a daily dose of 300 mg and reduced mortality [20]. Conversely, an RCT of low-dose fluvoxamine [21] (i.e., 100 mg/day) among overweight or obese outpatients with COVID-19 showed no significant benefit on emergency department visits, hospitalizations or death. These findings suggest that the use of fluvoxamine, when prescribed at a daily dose of 200–300 mg, may improve the clinical outcomes of patients infected with SARS-CoV-2, including mortality, in both ambulatory and acute care settings.

The benefits of fluvoxamine as a treatment for COVID-19 are believed to stem from several mechanisms [13, 18, 19, 22–27]. They include antiviral and anti-inflammatory effects via functional inhibition of acid sphingomyelinase (FIASMA), immunomodulatory activity via sigma-1 receptor (S1R) agonism and non-S1R pathways (e.g., NF-κB, inflammasomes, TLR4, PPARγ), and mechanisms related to increased plasma melatonin levels, serotonin modulation, and antiplatelet activity. Specifically, before the COVID-19 pandemic, fluvoxamine showed anti-inflammatory properties in murine models of septic shock [28], which may be mediated by the S1R pathway of cytokine release [24, 25, 29, 30]. Another mechanism through which the beneficial effects of fluvoxamine could be exerted is the acid sphingomyelinase/ceramide (ASM) pathway [27, 31–33]. Inhibition of the ASM/ceramide system (called FIASMA) by specific antidepressants such as fluvoxamine or fluoxetine prevents infection of Vero E6 cells with SARS-CoV-2 [23]. The reconstitution of ceramides in cells treated with these specific antidepressants restores the infection [23]. Furthermore, inhibition of ASM in endothelial cells and the immune system may also result in anti-inflammatory effects [9]. These data were reinforced in several other studies. First, several observational cohort studies of patients with COVID-19 reported reduced deaths or use of mechanical ventilation in the acute care setting [33–37] and reduced risk of emergency department or hospital visits in the ambulatory setting [38] among those taking FIASMA antidepressants versus their counterparts. Second, preclinical studies have demonstrated the in vitro efficacy of several SSRI and non-SSRI FIASMA antidepressants against different variants of SARS-CoV-2 in human and nonhuman host cells [22, 39–45]. Finally, a recent work [46] supported the antiviral and anti-inflammatory properties of fluoxetine, an SSRI antidepressant with FIASMA properties, in a K18-hACE2 mouse model of SARS-CoV-2 infection and its in vitro antiviral activity against different variants of concern, including Omicron BA.5.

Due to limited access to effective COVID-19 therapeutics, especially in most low- and middle-income countries, the promising data on fluvoxamine, coupled with its low cost, known good tolerability [47], and wide availability, support the importance of prospective interventional studies on fluvoxamine for outpatient and inpatient therapy of COVID-19 [9–11].

To evaluate the efficacy and tolerability of fluvoxamine in terms of mortality among inpatients with COVID-19, we performed a prospective interventional open-label cohort study. This study was performed during the third wave of SARS-CoV-2 infections in Uganda, marked by the Omicron variant.

## Materials and methods

### Design and patients

We prospectively included all COVID-19 inpatients treated at the COVID-19 treatment unit (CTU) of Mulago Hospital in Uganda from December 2021 to February 2022. We collected data on clinical characteristics, comorbidities, coprescribed treatments, disease severity, and treatment outcomes (i.e., all-cause mortality, duration of hospitalization and complete symptom resolution). The details of the Mulago Hospital CTU have been previously described [48]. Briefly, to be admitted or treated for COVID-19, patients had to have either a positive SARS-CoV-2 reverse transcriptase-polymerase chain reaction (RT‒PCR) test or a positive COVID-19 rapid antigen SARS-CoV-2 test (COVID-19 RDT) or clinical signs and radiological tests consistent with a COVID-19 diagnosis. After evaluation for any specific medical contraindication or expected deleterious drug‒drug interaction [49], fluvoxamine was offered at the discretion of the physician at a dose of 100 mg twice a day for 10 days (based on the dose and duration used in the TOGETHER trial [18]), in addition to standard care, to all patients who consented to compassionate use. The exclusion criteria included age less than 18 years and unstable medical comorbidities as judged by the admitting physician, including severe underlying lung disease (i.e., chronic obstructive pulmonary disease (COPD), interstitial lung disease and pulmonary hypertension), decompensated cirrhosis, congestive heart failure (New York Heart Association (NYHA) stage C or D), solid organ transplant, psychiatric disorders with behavioral problems, or increased risk of bleeding. All patients were followed up during hospitalization until discharge or death, and no patients were lost to follow-up. All patients (taking or not taking fluvoxamine) were monitored by the treating physician for FDA-listed known [49] or potentially unknown side effects of fluvoxamine.

### Patient management

Patients were treated according to national guidelines for the diagnosis and management of COVID-19 [50], as detailed in the Supplementary Text.

### Main and secondary outcomes

The study baseline was defined as the date of hospital admission. The end of the study was death or discharge. The main outcome was all-cause mortality. Secondary outcomes were hospital discharge and complete symptom resolution, which was defined as having none of the COVID-19 symptoms that were present at admission, based on patient self-reports.

### Statistical analysis

We used percentages for categorical variables and medians (interquartile ranges (IQRs)) for continuous variables to describe patients’ sociodemographic, COVID-19 symptom, vaccination status, comorbidity, and disease severity characteristics at admission. These characteristics were compared between patients taking versus those not taking fluvoxamine using standardized mean differences (SMDs) [51]. To examine the associations of fluvoxamine with the main outcome (all-cause mortality) and the two secondary outcomes (discharge and complete symptom resolution), we first performed univariate comparisons using SMDs for all outcomes as well as chi-squared tests (*χ*^2^) for binary outcomes and two-sample Mood’s median tests for continuous outcomes.

Next, we used time-to-event analyses adjusted for baseline sociodemographic and clinical characteristics and performed Cox proportional hazards regression models to examine the association of fluvoxamine with all-cause mortality and hospital discharge. To help account for the nonrandomized prescription of fluvoxamine and reduce the effects of confounding, the primary analyses used a propensity score analysis with inverse probability weighting (IPW) [52–55]. The individual propensities for exposure to fluvoxamine were estimated by multivariable logistic regression models that included all baseline characteristics (listed in Table 1 and in the footnotes of Table 2). In the IPW analyses, the predicted probabilities from the propensity score models were used to calculate the stabilized inverse probability weights [52, 53]. Associations of fluvoxamine with both outcomes were then estimated using IPW Cox regression models. In the case of nonbalanced covariates, IPW multivariable Cox regression models adjusting for the nonbalanced covariates were also performed. All Cox regression models estimating the association of fluvoxamine use with mortality were right-censored at the time of discharge. Kaplan‒Meier curves were drawn using the inverse probability weights [56], and their pointwise 95% confidence intervals were estimated using the nonparametric bootstrap method [57]. To examine the association of fluvoxamine with complete symptom resolution, we performed a multivariable logistic regression model that adjusted for all baseline characteristics and duration of follow-up and considered death as a failure to resolve symptoms. For all analyses, in the case of outliers, defined as values outside the 1.5 interquartile range (IQR), additional analyses excluding these outliers were also performed.

**Table 1:** Clinical characteristics of participants treated and not treated with fluvoxamine (*N* = 316).

	Total	Fluvoxamine	No fluvoxamine	Fluvoxamine versus No fluvoxamine			
	*N* = 316	*N* = 94	*N* = 222	Full sample^a^		IPW^b^	
	*N* (%)	*N* (%)	*N* (%)	*χ*^2^ (*p* value)/*Z* (*p* value)	SMD	*χ*^2^ (*p* value)/*Z* (*p* value)	SMD
Characteristics							
Age (years)—Median (IQR)	60.0 (37.0)	60.0 (32.0)	59.5 (39.5)	<0.01 (0.96)	0.014	1.09 (0.28)	0.062
Sex				<0.01 (>0.99)	0.003	0.01 (0.50)	0.099
Male	151 (47.8)	45 (47.9)	106 (47.7)				
Female	165 (52.2)	49 (52.1)	116 (52.3)				
COVID-19 symptoms							
Any symptom	297 (94.0)	92 (97.9)	205 (92.3)	2.67 (0.10)	0.258	2.78 (0.23)	0.262
Fever	76 (24.1)	30 (31.9)	46 (20.7)	3.94 (0.04)	0.256	2.06 (0.18)	0.068
Cough	253 (80.1)	79 (84.0)	174 (78.4)	1.00 (0.32)	0.145	2.32 (0.37)	0.069
Dyspnea	173 (54.7)	47 (50.0)	126 (56.8)	0.96 (0.33)	0.136	1.67 (0.56)	0.058
Muscle ache	26 (8.23)	8 (8.51)	18 (8.11)	<0.01 (>0.99)	0.015	0.26 (0.13)	0.085
Delirium	6 (1.90)	1 (1.06)	5 (2.25)	0.07 (0.80)	0.093	0.38 (0.23)	0.019
Headache	50 (15.8)	17 (18.1)	33 (14.9)	0.30 (0.58)	0.087	0.80 (0.89)	0.062
Pharyngitis	10 (3.16)	1 (1.06)	9 (4.05)	1.07 (0.30)	0.190	0.19 (0.77)	0.027
Rhinorrhea	6 (1.90)	1 (1.06)	5 (2.25)	0.07 (0.80)	0.093	0.37 (0.21)	0.065
Chest pain	84 (26.6)	33 (35.1)	51 (23.0)	4.38 (0.04)	0.270	1.26 (0.67)	0.037
Diarrhea	7 (2.22)	3 (3.19)	4 (1.80)	0.12 (0.73)	0.089	1.26 (0.63)	0.003
Nausea or vomiting	7 (2.22)	1 (1.06)	6 (2.70)	0.24 (0.63)	0.121	0.49 (0.11)	0.063
Vital signs							
Temperature (°C)—Median (IQR)^c^	37.2 (2.0)	37.7 (1.78)	37.1 (2.1)	2.71 (0.10)	0.168	0.48 (0.64)	0.121
Respiratory rate (breaths per minute)—Median (IQR)	20.0 (32.5)	19.0 (31.0)	29.8 (33.0)	0.38 (0.71)	0.018	1.51 (0.13)	0.175
Pulse rate (bpm)—Median (IQR)	92.0 (29.0)	87.0 (23.8)	96.0 (30.8)	7.67 (<0.001)	0.293	0.98 (0.32)	0.026
Blood pressure (mm Hg)				0.25 (0.25)	0.157	1.49 (0.29)	0.005
≤130/90	218 (69.0)	60 (63.8)	158 (71.2)				
>130/90	98 (31.0)	34 (36.2)	64 (28.8)				
Comorbidities							
Any comorbidity	153 (48.4)	46 (48.9)	107 (48.2)	<0.01 (>0.99)	0.015	0.88 (0.24)	0.029
Tuberculosis	11 (3.48)	0 (0.00)	11 (4.95)	3.46 (0.06)	0.323	3.21 (0.001)	0.227
Heart disease (CVD)	49 (15.5)	17 (18.1)	32 (14.4)	0.43 (0.51)	0.010	1.06 (0.81)	0.030
Asthma	8 (2.53)	3 (3.19)	5 (2.25)	0.01 (0.93)	0.058	0.68 (0.48)	0.021
COPD	3 (0.95)	2 (2.13)	1 (0.45)	0.59 (0.44)	0.149	3.60 (0.68)	0.030
Diabetes	83 (26.3)	25 (26.6)	58 (26.1)	<0.01 (>0.99)	0.011	0.01 (0.36)	0.096
Cancer	8 (2.53)	1 (1.06)	7 (3.15)	0.47 (0.49)	0.146	1.19 (0.31)	0.113
HIV	42 (13.3)	9 (9.57)	33 (14.9)	1.18 (0.28)	0.162	2.07 (0.35)	0.015
Oxygen therapy at admission				14.76 (0.001)	0.462	2.18 (0.72)	0.032
No supplemental O_2_	237 (75.0)	57 (60.6)	180 (81.1)				
<10 l/min	50 (15.8)	23 (24.5)	27 (12.2)				
10+ l/min	29 (9.18)	14 (14.9)	15 (6.76)				
Vaccination status				2.71 (0.26)	0.193	3.13 (0.36)	0.047
At least 1 dose of COVID-19 vaccine	81 (25.6)	29 (30.9)	52 (23.4)				
1 dose	33 (10.4)	10 (10.6)	23 (10.4)				
2 doses	48 (15.2)	19 (20.2)	29 (13.1)				
Not vaccinated	235 (74.4)	65 (69.1)	170 (76.6)				
Co-prescribed COVID-19 medications							
Dexamethasone	232 (73.4)	70 (74.5)	162 (73.0)	0.02 (0.89)	0.034	0.05 (0.65)	0.038
Inhaled budesonide	16 (5.06)	7 (7.45)	9 (4.05)	0.95 (0.33)	0.146	2.38 (0.69)	<0.001
Antibiotics	159 (50.3)	48 (51.1)	111 (50.0)	<0.01 (0.96)	0.021	0.13 (0.16)	0.120
COVID-19 diagnostic methods				<0.01 (0.99)	0.059	0.64 (0.62)	0.017
Positive RT-PCR	5 (1.58)	2 (2.13)	3 (1.35)				
Positive RDT	311 (98.4)	92 (97.9)	219 (98.6)				

*CVD* cardiovascular disease, *COPD* chronic obstructive pulmonary disease, *RDT* antigen detection rapid diagnostic test, *bpm* beats per minute.

^a^We used chi-squared tests (*χ*^2^) or Fisher’s exact tests if necessary for categorical variables and two-sample Mood’s median tests for continuous variables.

^b^We used weighted chi-squared tests (*χ*^2^) for categorical variables and weighted two-sample Mood’s median tests for continuous variables.

^c^Temperature was measured using the axillary route.

**Table 2:** Association of fluvoxamine with all-cause mortality and complete symptom resolution and, among survivors, with hospital discharge.

	Patients with fluvoxamine	Patients without fluvoxamine	Crude Cox regression analysis	Multivariable Cox regression analysis^a^	Cox regression analysis weighted by IPW^b^	Cox regression analysis weighted by IPW adjusted for unbalanced covariates	Cox regression analysis weighted by IPW adjusted for unbalanced covariates after excluding outliers
	Events/*N* (%)	Events/*N* (%)	HR (95% CI; *p* value)	AHR (95% CI; *p* value)	HR (95% CI; *p* value)	AHR (95% CI; *p* value)	AHR (95% CI; *p* value) [number of outliers]
Mortality							
Fluvoxamine	29/94 (30.9)	126/222 (56.8)	0.44 (0.30–0.67; <0.001)	0.29 (0.18–0.47; <0.001)	0.33 (0.15–0.72; 0.005)	0.32 (0.19–0.53; <0.001)^c^	0.34 (0.23–0.51; <0.001) [9]^f^
Hospital discharge among survivors							
Fluvoxamine	65/65 (100)	96/96 (100)	0.86 (0.62–1.18; 0.34)	0.89 (0.58–1.37; 0.60)	0.81 (0.54–1.23; 0.32)	0.86 (0.59–1.25; 0.43)^d^	0.87 (0.60–1.27; 0.46) [8]^g^
	Patients with fluvoxamine	Patients without fluvoxamine	Crude logistic regression analysis	Multivariable logistic regression analysis^a^	Multivariable logistic regression analysis^e^		Multivariable logistic regression analysis after excluding outliers^e^
	Events/*N* (%)	Events/*N* (%)	OR (95% CI; *p* value)	AOR (95% CI; *p* value)	AOR (95% CI; *p* value)		AOR (95% CI; *p* value)
Complete symptom resolution							
Fluvoxamine	51/94 (54.3)	69/222 (31.1)	2.63 (1.61–4.33; <0.001)	2.56 (1.53–5.51; 0.001)	2.13 (1.03–4.40; 0.04)		3.30 (1.45–7.71; 0.005) [11]^h^

^a^Adjusted for age, sex, fever, cough, dyspnea, muscle ache, delirium, headache, pharyngitis, rhinorrhea, chest pain, diarrhea, and nausea or vomiting, temperature, respiratory rate, pulse rate, blood pressure, tuberculosis, heart disease, asthma, COPD, diabetes, cancer, HIV, oxygen therapy at admission, vaccination status, dexamethasone, inhaled budesonide, antibiotics, and method of COVID-19 diagnosis (degrees of freedom (df) = 31, all generalized variance inflation factors (GVIFs) <1.9).

^b^The individual propensities for exposure to fluvoxamine were estimated by multivariable logistic regression models that included age, sex, fever, cough, dyspnea, muscle ache, delirium, headache, pharyngitis, rhinorrhea, chest pain, diarrhea, and nausea or vomiting, temperature, respiratory rate, pulse rate, blood pressure, tuberculosis, heart disease, asthma, COPD, diabetes, cancer, HIV, oxygen therapy at admission, vaccination status, dexamethasone, inhaled budesonide, antibiotics, and method of COVID-19 diagnosis (df = 31).

^c^Adjusted for temperature, respiratory rate, tuberculosis, cancer, and antibiotics.

^d^Adjusted for muscle ache, delirium, pharyngitis, pulse rate, tuberculosis, COPD, cancer, and HIV.

^e^Adjusted for age, sex, fever, cough, dyspnea, muscle ache, delirium, headache, pharyngitis, rhinorrhea, chest pain, diarrhea, and nausea or vomiting, temperature, respiratory rate, pulse rate, blood pressure, tuberculosis, heart disease, asthma, COPD, diabetes, cancer, HIV, oxygen therapy at admission, vaccination status, dexamethasone, inhaled budesonide, antibiotics, method of COVID-19 diagnosis, and duration of follow-up (df = 32, all GVIF < 1.8).

^f^Fluvoxamine: Events/*N* (%) = 29/93 (31.2%); No fluvoxamine: Events/*N* (%) = 123/214 (57.5%).

^g^Fluvoxamine: *N* = 61; No fluvoxamine: *N* = 92.

^h^Fluvoxamine: Events/*N* (%) = 50/90 (55.6%); No fluvoxamine: Events/*N* (%) = 63/215 (29.3%).

We performed two sensitivity analyses. First, we used nearest neighbor matching to obtain a matched analytic sample [58] using a 1:1 ratio in the full sample and in the subsample of survivors, based on the same variables used for the IPW analysis. In these matched samples, we performed univariate Cox regression models for mortality and discharge and a univariate logistic regression model for complete symptom resolution. In the case of nonbalanced covariates, multivariable regression models adjusting for the nonbalanced covariates were also performed. Second, we reproduced the main analysis for mortality while censoring at day 15 and 28.

In the case of a significant association between fluvoxamine use and any outcome in the primary IPW analysis, we planned to calculate the between-group difference in absolute risk reduction/increase (ARR/ARI) [59] and number needed to treat (NNT) [59, 60] while taking into account the weighted time-to-event design when used and to perform post hoc exploratory analyses evaluating interactions of fluvoxamine with each baseline characteristic (e.g., vaccination status, baseline oxygen level) to assess their potential impact on the association between the outcome and fluvoxamine use.

The rates of adverse events of fluvoxamine [49] were also calculated and compared between inpatients with COVID-19 who did and did not receive the treatment.

For all associations, we performed residual analyses to assess the fit of the data, checked assumptions, including the proportional hazards assumption, using proportional hazards tests and diagnostics based on weighted residuals [61, 62], and examined the potential influence of outliers. We followed the recommendations of the STROBE reporting guideline for cohort studies. All analyses were performed using *R* software, version 4.2.1 [63]. Statistical significance was fixed a priori at a two-sided *p* value <0.05.

## Results

### Clinical characteristics

**Fig. 1:**
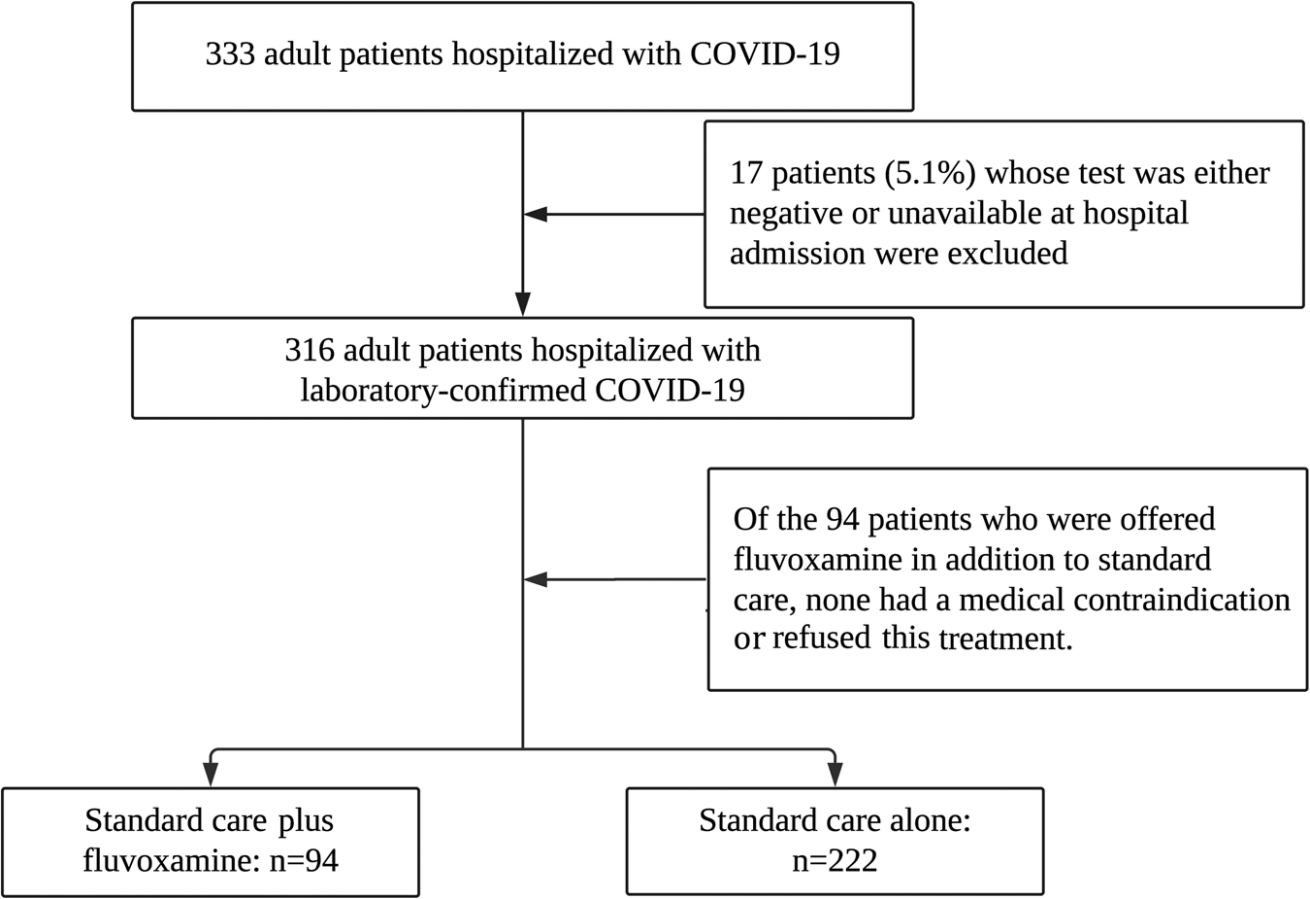
Study cohort. Study flow diagram showing participant recruitment.

Of the 333 adult inpatients with COVID-19 treated at the CTU of Mulago Hospital, 17 (5.1%) patients were excluded because their COVID-19 biological test was either negative or unavailable at admission (Fig. 1). Of the remaining 316 patients, 94 (29.7%) were proposed fluvoxamine in addition to standard care, and all these patients accepted it. The median age of the patients was 60.0 years (IQR = 37.0), and 52.2% were women. The participants’ characteristics are summarized in Table 1. Compared with those without fluvoxamine, patients with fluvoxamine were more likely to have COVID-19 symptoms, COPD, oxygen therapy at admission, at least one dose of COVID-19 vaccine, coprescribed inhaled budesonide, higher temperature and blood pressure, and lower pulse rate; they were less likely to have tuberculosis, cancer, and HIV (Table 1). After applying the propensity score weights, these differences according to fluvoxamine exposure were reduced (Table 1). The distribution of characteristics in the subsample of survivors and in the matched analytic samples is given in eTables 1–3. The distribution of the inverse propensity score weights is given in eFigs. 1 and 2.

### Effectiveness of fluvoxamine

Over a median follow-up of 5 days (IQR = 5 days) until discharge or death, 155 patients (49.05%) died. A total of 29 patients (30.9%) who took fluvoxamine and 126 patients (56.8%) who did not died during hospitalization. Univariate comparison of mortality rates indicated a significantly lower mortality rate among patients who received fluvoxamine versus those who did not (SMD = 0.53; *χ*^2^ = 16.71, *p* < 0.001). Univariate comparison of complete symptom resolution indicated a significantly higher rate of symptom resolution among patients who received fluvoxamine versus those who did not [fluvoxamine group: 51/94 (54.3%); comparison group: 69/222 (31.1%); SMD = 0.49; *χ*^2^ = 14.09, *p* < 0.001]. Among the 161 survivors, univariate comparison of the median time from hospital admission to discharge did not show a significant difference [fluvoxamine group: median (IQR) = 7 (6); comparison group: median (IQR) = 6 (6); SMD = 0.27; two-sample Mood’s median test = 0.30, *p* = 0.76].

**Fig. 2:**
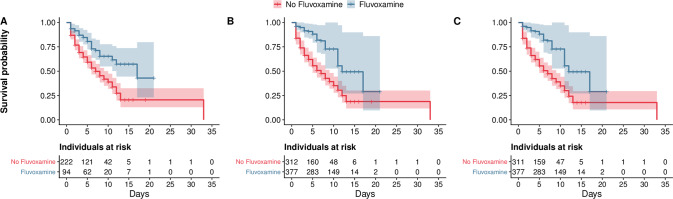
Kaplan-Meier curves for all-cause mortality. The shaded areas represent point wise 95% CI. **A** Kaplan-Meier curves for all-cause mortality in the crude analysis. **B** Kaplan-Meier curves for all-cause mortality in the analysis with inverse probability weighting (IPW). **C** Kaplan-Meier curves for all-cause mortality in the analysis with IPW while excluding outliers.

In the full sample of 316 inpatients, the unadjusted analysis (hazard ratio (HR) = 0.44; 95% CI = 0.30–0.67; *p* < 0.001), multivariable Cox regression analysis (AHR = 0.29; 95% CI = 0.18–0.47; *p* < 0.001), primary analysis with IPW (HR = 0.33; 95% CI = 0.15–0.72; *p* = 0.005), multivariable IPW Cox regression adjusting for unbalanced variables (AHR = 0.32; 95% CI = 0.19–0.53; *p* < 0.001), and multivariable IPW Cox regression excluding outliers (AHR = 0.34; 95% CI = 0.23–0.51; *p* < 0.001) showed a significant and substantial association between fluvoxamine use and reduced mortality (Table 2 and Fig. 2), corresponding to an ARR of death of 22.4% and an NNT of 4.46.

Fluvoxamine was significantly associated with greater rates of complete symptom resolution in the fully adjusted multivariable logistic regression model (AOR = 2.13; 95% CI = 1.03–4.40; *p* = 0.04), corresponding to an ARI of 23.2% and an NNT of 4.44, excluding outliers (AOR = 3.30; 95% CI = 1.45–7.71; *p* = 0.005).

Among survivors, fluvoxamine was not significantly associated with time to hospital discharge in the IPW Cox regression models (AHR = 0.86; 95% CI = 0.59–1.25; *p* = 0.43) or in the IPW Cox regression models that excluded outliers (AHR = 0.87; 95% CI = 0.60–1.27; *p* = 0.46) (Table 2 and eFig. 3).

In sensitivity analyses, the univariate Cox regression models in the 1:1 ratio matched analytic samples showed similar results (eTable 4 and eFig. 4), as did the primary analyses for mortality while censoring at day 15 and 28 (eTable 5).

Exploratory analyses suggested that the associations of fluvoxamine with mortality and complete symptom resolution did not significantly differ according to participants’ characteristics (eTables 6 and 7). In particular, the association of fluvoxamine with mortality was significant among both nonvaccinated patients and patients who had received at least 1 dose of the COVID-19 vaccine.

### Tolerability of fluvoxamine

The frequency of side effects, mostly light or mild, was higher, albeit not significantly, among participants who received fluvoxamine (7.45%, *N* = 7) than among those who did not (3.15%, *N* = 7) (SMD = 0.21, *χ*^2^ = 3.46, *p* = 0.06) (Table 3).

**Table 3:** Frequency of adverse events among participants treated and not treated with fluvoxamine (*N* = 316).

Adverse events	Fluvoxamine (*n* = 94)	No fluvoxamine (*n* = 222)
	*N* (%)	*N* (%)
Nausea	0 (0.0)	1 (0.5)
Vomiting	0 (0.0)	1 (0.5)
Abdominal pain	0 (0.0)	1 (0.5)
Constipation	0 (0.0)	0 (0.0)
Diarrhea	0 (0.0)	0 (0.0)
Appetite loss	3 (3.2)	0 (0.0)
Xerostomia	0 (0.0)	0 (0.0)
Drowsiness	0 (0.0)	0 (0.0)
Insomnia	0 (0.0)	0 (0.0)
Dizziness	0 (0.0)	1 (0.5)
Nervousness	0 (0.0)	0 (0.0)
Anxiety	0 (0.0)	0 (0.0)
Headache	3 (3.2)	1 (0.5)
Muscle weakness	0 (0.0)	0 (0.0)
Paresthesia	0 (0.0)	0 (0.0)
Dysgeusia	0 (0.0)	0 (0.0)
Tachycardia	0 (0.0)	0 (0.0)
Hyperhidrosis	0 (0.0)	0 (0.0)
Syncope	0 (0.0)	0 (0.0)
Seizure	0 (0.0)	0 (0.0)
Glaucoma	0 (0.0)	0 (0.0)
Blurred vision	0 (0.0)	0 (0.0)
Back pain	0 (0.0)	1 (0.5)
Cough	1 (1.1)	0 (0.0)
Delirium	0 (0.0)	1 (0.5)
At least one of above	7 (7.5)	7 (3.2)

## Discussion

This prospective, interventional, open-label cohort study was a real-world evidence study supporting that 100 mg of fluvoxamine prescribed twice a day for 10 days in addition to standard care was significantly associated with reduced mortality and increased complete symptom resolution compared with standard care alone among adult patients hospitalized for COVID-19 in Uganda. These associations did not significantly differ according to clinical characteristics, including vaccination status. The frequency of side effects, mostly light or mild, was relatively low but higher, albeit not significantly, among participants who received fluvoxamine versus those who did not. Among survivors, no significant difference was found in terms of the duration of hospitalization.

These findings are in many ways consistent with prior preclinical and observational research findings suggesting that certain antidepressants, including fluvoxamine, could be beneficial against COVID-19 [9, 13, 17, 19, 23, 25, 33–36, 64, 65]. They are also consistent with the findings of three studies, including two RCTs and one nonrandomized open-label clinical study, which found a significant association between the short-term use (10–15 days) of fluvoxamine within 7 days of symptom onset and a reduced risk of clinical deterioration among outpatients with COVID-19 [17–19]. Specifically, in the STOP-COVID trial, clinical deterioration occurred in 0 of 80 patients in the fluvoxamine group and in 6 of 72 patients in the placebo group (absolute difference, 8.7% [95% CI, 1.8–16.4%] log-rank *p* = 0.009) [19]. The TOGETHER trial performed in an ambulatory setting reported a significantly lower risk of emergency department retention >6 h or hospital admission with fluvoxamine use compared with placebo [79 [11%] of 741 versus 119 [16%] of 756; relative risk [RR] 0.68; 95% Bayesian credible interval [95% BCI] = 0.52–0.88] [18, 66]. Three recent systematic reviews and meta-analyses reported that fluvoxamine significantly and substantially reduced hospitalization risk among outpatients with COVID-19 [13–16]. Finally, a prospective cohort study of patients admitted to the ICU for COVID-19 reported a significant association between the 15-day use of fluvoxamine and reduced mortality [20].

An RCT of fluvoxamine [21] prescribed at 100 mg/day among overweight and obese outpatients with COVID-19 showed no significant benefit on the risk of emergency department visits, hospitalizations or death, contrasting with the positive findings of TOGETHER and STOP-COVID, in which fluvoxamine was prescribed at doses of 200 and 300 mg/day, respectively. This discrepancy may be explained by a potential effect of fluvoxamine that occurs at a minimum dose of 200 mg/d, as suggested by two observational studies [36, 38] that found that exposure to antidepressants, especially those with FIASMA properties such as fluvoxamine, was associated with a reduced incidence of emergency department visits or hospital admissions among SARS-CoV-2-positive outpatients and with reduced 28-day mortality among COVID inpatients in a dose-dependent manner and from daily doses of at least 20 mg of fluoxetine equivalents. These findings suggest that fluvoxamine should be prescribed at a minimum daily dose of 200 mg (100 mg twice daily) to observe a benefit in patients with COVID-19, as was done in this study.

Overall, a relatively small proportion (7.45%) of patients who took fluvoxamine for 10 days reported side effects, mostly light or mild symptoms known to occur with fluvoxamine, and none of them were serious, with transient headache and appetite loss being the most common symptoms encountered, which is also consistent with COVID-19 symptoms. The frequency of side effects was nonetheless higher, albeit not significantly, in the fluvoxamine group.

The main strength of this study is that we provide for the first time, to our knowledge, real-world data on the efficacy and tolerability of fluvoxamine among inpatients with COVID-19 in Africa. However, this study has several limitations. First, because of the study design (nonrandomized and unblinded study), biases may have occurred at different levels. However, the use of mortality as the main outcome and the congruence of our observations with the findings of other studies, including a large randomized, placebo-controlled, prospective trial among high-risk outpatients [18], reduce this concern. Furthermore, we used different statistical approaches and sensitivity analyses that yielded similar results, suggesting the robustness of our results. Second, we did not collect data on viral clearance or inflammatory markers, preventing us from assessing the mechanisms through which fluvoxamine confers benefits against COVID-19. Third, the use of the exclusion criterion “unstable medical comorbidities” as judged by the admitting physician instead of a more objective measure might have introduced a potential bias. Fourth, data on potential deaths after hospital discharge were not available. Finally, we were not able to determine treatment adherence or potential unexpected drug‒drug interactions through the blood dosage of fluvoxamine.

In conclusion, this study confirms and extends prior RCT results in finding that fluvoxamine prescribed at 100 mg twice a day is well tolerated and significantly associated with reduced mortality and increased complete symptom resolution among COVID-19 inpatients. RCTs of fluvoxamine prescribed at a daily dose of 200 mg are urgently needed to confirm these results among inpatients with COVID-19.

## Supplementary information

Supplementary material

## Data Availability

All data and the R code used for this study are available at https://github.com/mlsrico/fluvoxamine_uganda.
