# Supplementary material for: Association of fluvoxamine with mortality and symptom resolution among inpatients with COVID-19 in Uganda: a prospective interventional open-label cohort study
Source: Mol Psychiatry. 2023 Mar 3;28(12):5411–8. doi: 10.1038/s41380-023-02004-3 (PMC9982784; doi:10.1038/s41380-023-02004-3)
Supplement: Supplementary file 1 — Supplementary material [file 41380_2023_2004_MOESM1_ESM.docx]

**SUPPLEMENTARY MATERIAL**

**Supplementary Text**

**Patient management**

Patients were treated according to the National COVID-19 case management guidelines. Briefly, patients with mild-moderate disease benefited from zinc supplements. Those with respiratory symptoms in whom superimposed bacterial infection was diagnosed or suspected were treated with antibiotics according to the physician’s judgement. Patients with severe-critical disease were treated with intravenous dexamethasone and anticoagulant therapy. Other treatments were used according to the complications the patients presented with and according to the attending physician’s judgement. Patients who required oxygen therapy received it according to their oxygen demand, i.e., by nasal cannula, face mask, high-flow nasal cannula (HFNC), noninvasive ventilation (NIV), continuous positive airway pressure (CPAP), or invasive mechanical ventilation (IMV).

**eFigure 1. Distribution of inverse probability score weights among inpatients with COVID-19 who did and did not receive fluvoxamine in the full sample (A) and among survivors (B).** The weights were truncated by resetting the value of weights greater than percentile 99 to the value of percentile 99.


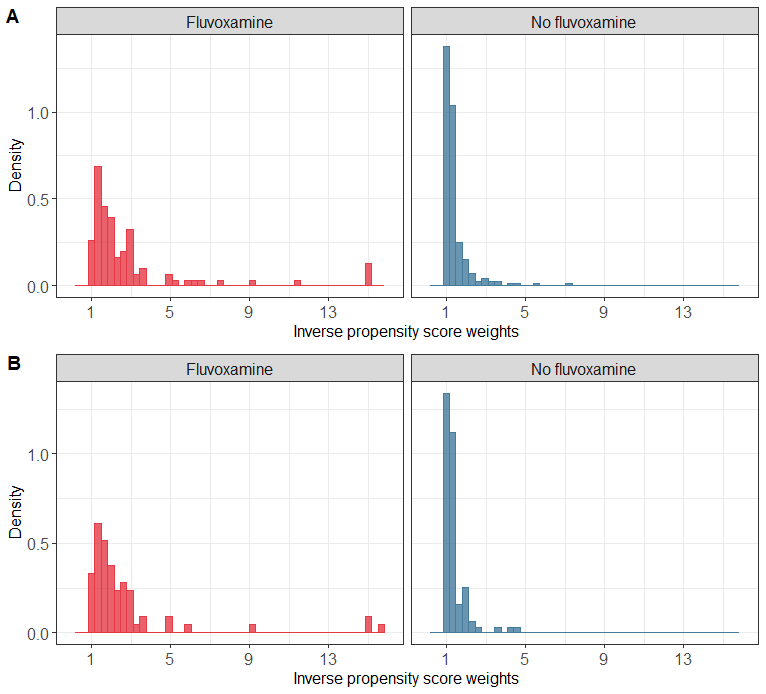


**eFigure 2. Distribution of the estimated propensity score for receiving fluvoxamine among inpatients with COVID-19 who did and did not receive fluvoxamine.**


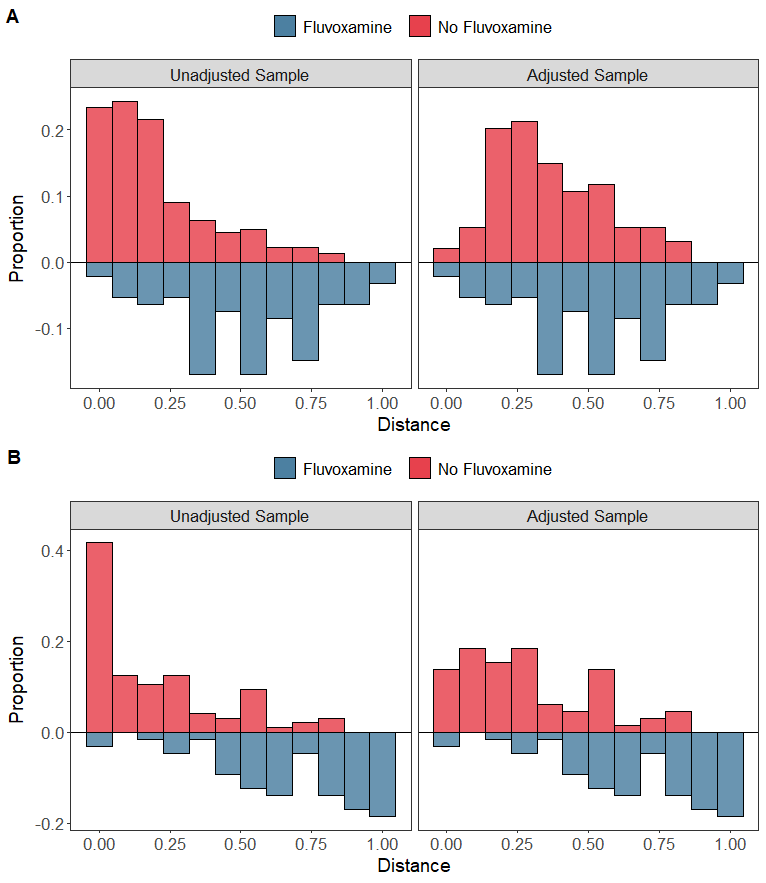


**eFigure 3. Kaplan-Meier curves for hospital discharge among those who survived in the crude analysis (A), in the analysis with inverse probability weighting (IPW) (B), and in the analysis with IPW excluding outliers (C), according to fluvoxamine use, among COVID-19 adult inpatients who survived.**

**
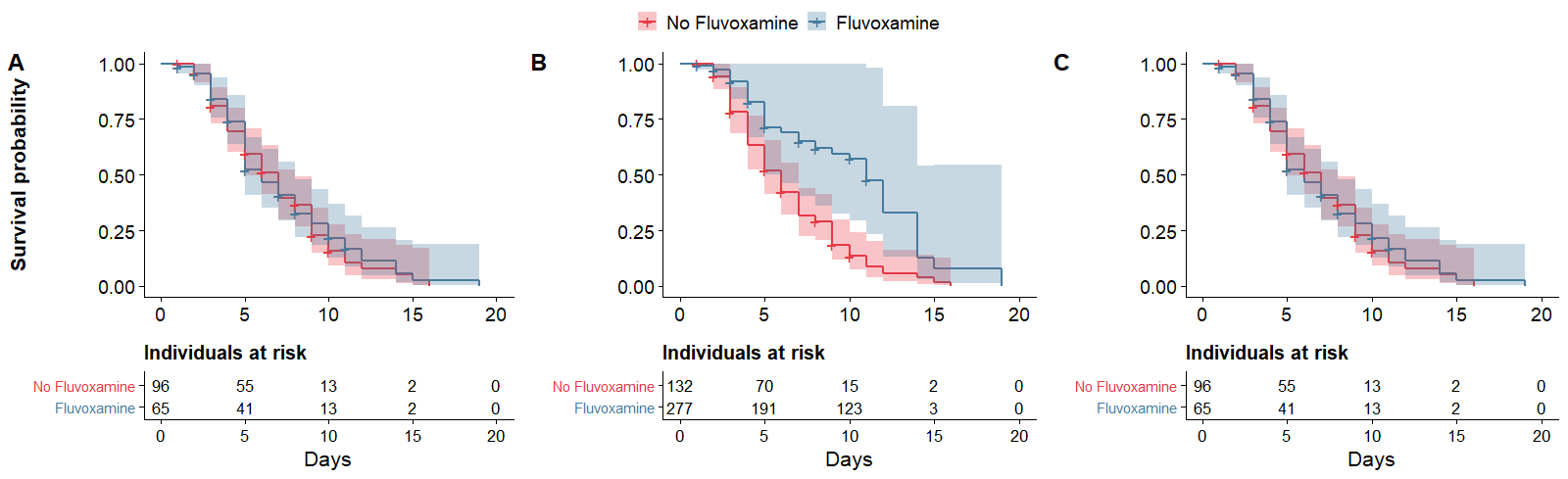
**

*Note:* The shaded areas represent pointwise 95% CIs. Numbers at risk in panels B and C are weighted.

**eFigure 4. Kaplan-Meier curves for mortality (A) and for hospital discharge among survivors (B) according to fluvoxamine use in the matched analytic samples of COVID-19 inpatients.**


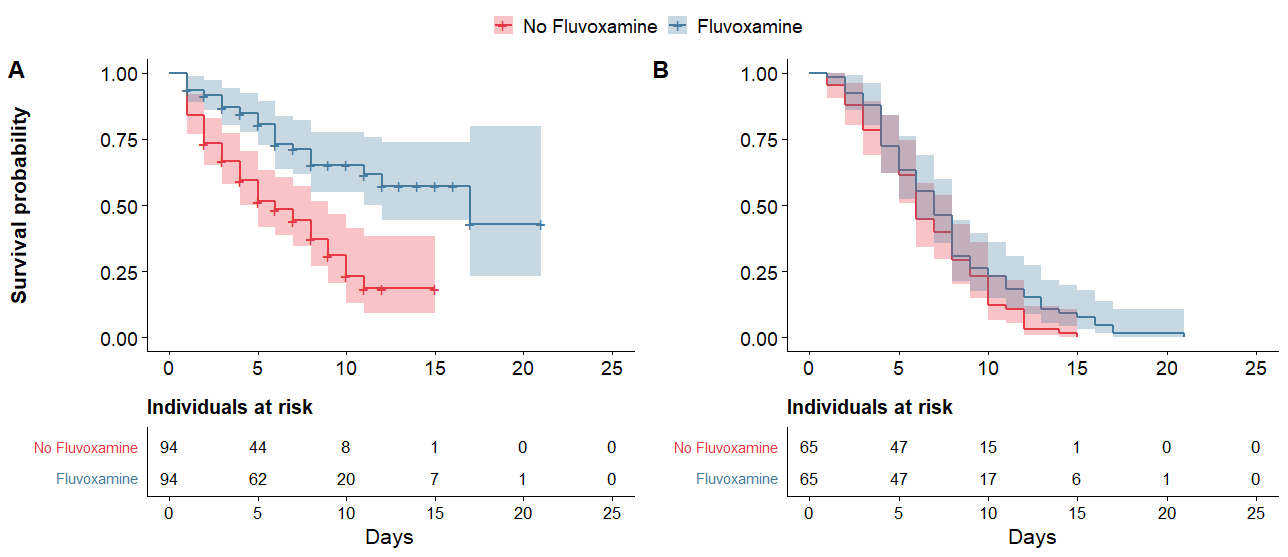


Note: The shaded areas represent pointwise 95% CIs.

**eTable 1. Clinical characteristics of participants treated and not treated with fluvoxamine who survived (N=161).**

|  | **Total** | **Fluvoxamine** | **No fluvoxamine** | **Fluvoxamine vs. No fluvoxamine** | | | |
| --- | --- | --- | --- | --- | --- | --- | --- |
|  | **N=161** | **N=65** | **N=96** | **Full sample ^a^** | | **IPW ^b^** | |
|  | **N (%)** | **N (%)** | **N (%)** | **χ^2^ (p-value) /**  **Z (p-value)** | **SMD** | **χ^2^ (p-value)/**  **Z (p-value)** | **SMD** |
| **Characteristics** |  |  |  |  |  |  |  |
| Age (years) - *Median (IQR)* | 51.9 (21.0) | 55.1 (20.9) | 49.7 (20.9) | -0.12 (0.91) | 0.258 | 0.43 (0.67) | 0.099 |
| Sex |  |  |  | 0.20 (0.65) | 0.098 | 0.71 (0.40) | 0.057 |
| *Male* | 79 (49.1) | 30 (46.2) | 49 (51.0) |  |  |  |  |
| *Female* | 82 (50.9) | 35 (53.8) | 47 (49.0) |  |  |  |  |
| **COVID-19 symptoms** |  |  |  |  |  |  |  |
| Any symptom | 150 (93.2) | 64 (98.5) | 86 (89.6) | 3.51 (0.06) | 0.381 | 2.32 (0.17) | 0.306 |
| Fever | 48 (29.8) | 24 (36.9) | 24 (25.0) | 2.09 (0.15) | 0.260 | <0.01 (0.97) | 0.026 |
| Cough | 122 (75.8) | 55 (84.6) | 67 (69.8) | 3.87 (0.05) | 0.359 | 0.84 (0.44) | 0.047 |
| Dyspnea | 67 (41.6) | 27 (41.5) | 40 (41.7) | <0.01 (>0.99) | 0.003 | 0.02 (0.92) | 0.006 |
| Muscle ache | 17 (10.6) | 6 (9.23) | 11 (11.5) | 0.04 (0.99) | 0.073 | 0.602 (0.43) | 0.114 |
| Delirium | 5 (3.11) | 0 (0.00) | 5 (5.21) | 1.98 (0.16) | 0.332 | 2.43 (0.12) | 0.203 |
| Headache | 38 (23.6) | 13 (20.0) | 25 (26.0) | 0.49 (0.49) | 0.144 | <0.01 (0.96) | 0.044 |
| Pharyngitis | 5 (3.11) | 0 (0.00) | 5 (5.21) | 1.98 (0.16) | 0.332 | 2.43 (0.12) | 0.203 |
| Rhinorrhea | 5 (3.11) | 1 (1.54) | 4 (4.17) | 0.23 (0.63) | 0.158 | 0.30 (0.60) | 0.089 |
| Chest pain | 45 (28.0) | 26 (40.0) | 19 (19.8) | 6.89 (0.009) | 0.453 | 0.17 (0.74) | 0.034 |
| Diarrhea | 4 (2.48) | 2 (3.08) | 2 (2.08) | <0.01 (>0.99) | 0.063 | 0.06 (0.81) | 0.025 |
| Nausea or vomiting | 3 (1.86) | 1 (1.54) | 2 (2.08) | <0.01 (>0.99) | 0.041 | 0.04 (0.84) | 0.033 |
| **Vital signs** |  |  |  |  |  |  |  |
| Temperature (°C) - *Median (IQR)* | 37.0 (1.11) | 37.1 (1.16) | 36.9 (1.08) | -0.54 (0.59) | 0.173 | -0.14 (0.89) | 0.023 |
| Respiratory rate (breaths *per* minute) - *Median (IQR)* | 15 (31.5) | 15 (31.5) | 15 (31.0) | <0.01 (>0.99) | 0.008 | 0.18 (0.86) | 0.033 |
| Pulse rate (bpm) - *Median (IQR)* | 94 (19.6) | 91 (20.0) | 96 (19.2) | -0.29 (0.77) | 0.266 | -0.80 (0.42) | 0.134 |
| Blood Pressure (mm Hg) |  |  |  | 0.01 (0.94) | 0.039 | 0.03 (0.89) | 0.049 |
| *≤130/90* | 91 (56.5) | 36 (55.4) | 55 (57.3) |  |  |  |  |
| *>130/90* | 70 (43.5) | 29 (44.6) | 41 (42.7) |  |  |  |  |
| **Comorbidities** |  |  |  |  |  |  |  |
| Any comorbidity | 79 (49.1) | 30 (46.2) | 49 (51.0) | 0.20 (0.65) | 0.098 | 0.26 (0.67) | 0.029 |
| Tuberculosis | 4 (2.48) | 0 (0.00) | 4 (4.17) | 1.32 (0.25) | 0.295 | 1.94 (0.16) | 0.181 |
| Heart disease (CVD) | 37 (23.0) | 15 (23.1) | 22 (22.9) | <0.01 (>0.99) | 0.004 | 0.02 (0.91) | 0.049 |
| Asthma | 4 (2.48) | 3 (4.62) | 1 (1.04) | 0.84 (0.36) | 0.217 | 0.50 (0.46) | 0.074 |
| COPD | 2 (1.24) | 2 (3.08) | 0 (0.00) | 1.01 (0.32) | 0.252 | 1.29 (0.14) | 0.115 |
| Diabetes | 41 (25.5) | 16 (24.6) | 25 (26.0) | <0.01 (0.98) | 0.033 | 0.32 (0.62) | 0.090 |
| Cancer | 6 (3.73) | 1 (1.54) | 5 (5.21) | 0.61 (0.43) | 0.204 | 0.79 (0.39) | 0.127 |
| HIV | 18 (11.2) | 2 (3.08) | 16 (16.7) | 5.90 (0.01) | 0.468 | 2.19 (0.29) | 0.185 |
| **Oxygen therapy at admission** |  |  |  | 6.77 (0.03) | 0.423 | 0.52 (0.83) | 0.082 |
| *No Supplemental O2* | 94 (58.4) | 30 (46.2) | 64 (66.7) |  |  |  |  |
| *<10 l/min* | 43 (26.7) | 22 (33.8) | 21 (21.9) |  |  |  |  |
| *10+ l/min* | 24 (14.9) | 13 (20.0) | 11 (11.5) |  |  |  |  |
| **Vaccination status** |  |  |  | 0.25 (0.88) | 0.078 | 0.10 (0.95) | 0.037 |
| *At least 1 dose of COVID-19 vaccine* | 51 (31.7%) | 22 (33.8%) | 29 (30.2%) |  |  |  |  |
| *1^st^ dose* | 19 (11.8) | 8 (12.3) | 11 (11.5) |  |  |  |  |
| *2^nd^ dose* | 32 (19.9) | 14 (21.5) | 18 (18.8) |  |  |  |  |
| *Not vaccinated* | 110 (68.3) | 43 (66.2) | 67 (69.8) |  |  |  |  |
| **Co-prescribed COVID-19 medications** |  |  |  |  |  |  |  |
| Dexamethasone | 102 (63.4) | 46 (70.8) | 56 (58.3) | 2.07 (0.15) | 0.262 | 0.28 (0.67) | 0.0580 |
| Inhaled budesonide | 5 (3.11) | 2 (3.08) | 3 (3.12) | <0.01 (>0.99) | 0.003 | <0.01 (0.97) | 0.0146 |
| Antibiotics | 107 (66.5) | 42 (64.6) | 65 (67.7) | 0.06 (0.81) | 0.065 | 0.17 (0.73) | 0.0669 |
| **COVID-19 diagnostic methods** |  |  |  | <0.01 (>0.99) | 0.063 | 0.01 (0.89) | 0.0092 |
| *Positive RT-PCR* | 4 (2.48) | 2 (3.08) | 2 (2.08) |  |  |  |  |
| *Positive RDT* | 157 (97.5) | 63 (96.9) | 94 (97.9) |  |  |  |  |

^a^ We used Chi-squared test (χ2) or Fisher’s exact tests if necessary for categorical variables and two-sample Mood’s median tests for continuous variables.

^b^ We used weighted Chi-squared test (χ2) for categorical variables and weighted two-sample Mood’s median tests for continuous variables.

**eTable 2. Clinical characteristics of participants treated and not treated with fluvoxamine in the 1:1 ratio matched analytic sample (N=188).**

|  | **Fluvoxamine**  **N=94** | **No fluvoxamine**  **N=94** | **Fluvoxamine vs. No fluvoxamine** | |
| --- | --- | --- | --- | --- |
|  | **N (%)** | **N (%)** | **χ^2^ (p-value) /**  **Z (p-value) ^a^** | **SMD** |
| **Characteristics** |  |  |  |  |
| Age (years) - *Median (IQR)* | 60.0 (32.0) | 58.0 (39.0) | -0.15 (0.88) | 0.018 |
| Sex |  |  | <0.01 (>0.99) | 0.021 |
| *Male* | 45 (47.9) | 44 (46.8) |  |  |
| *Female* | 49 (52.1) | 50 (53.2) |  |  |
| **COVID-19 symptoms** |  |  |  |  |
| Any symptom | 92 (97.9) | 91 (96.8) | <0.01 (>0.99) | 0.066 |
| Fever | 30 (31.9) | 24 (25.5) | 0.65 (0.42) | 0.141 |
| Cough | 79 (84.0) | 75 (79.8) | 0.32 (0.57) | 0.111 |
| Dyspnea | 47 (50.0) | 46 (48.9) | <0.01 (>0.99) | 0.021 |
| Muscle ache | 8 (8.51) | 6 (6.38) | 0.08 (0.78) | 0.081 |
| Delirium | 1 (1.06) | 1 (1.06) | <0.01 (>0.99) | <0.001 |
| Headache | 17 (18.1) | 12 (12.8) | 0.65 (0.42) | 0.148 |
| Pharyngitis | 1 (1.06) | 1 (1.06) | <0.01 (>0.99) | <0.001 |
| Rhinorrhea | 1 (1.06) | 2 (2.13) | <0.01 (>0.99) | 0.085 |
| Chest pain | 33 (35.1) | 29 (30.9) | 0.22 (0.64) | 0.091 |
| Diarrhea | 3 (3.19) | 1 (1.06) | 0.25 (0.61) | 0.148 |
| Nausea or vomiting | 1 (1.06) | 1 (1.06) | <0.01 (>0.99) | <0.001 |
| **Vital signs** |  |  |  |  |
| Temperature (°C) - *Median (IQR)* | 37.7 (1.78) | 37.3 (2.27) | -0.58 (0.56) | 0.009 |
| Respiratory rate (breaths *per* minute) - *Median (IQR)* | 19 (30.5) | 20 (31.5) | 0.87 (0.38) | 0.140 |
| Pulse rate (bpm) - *Median (IQR)* | 87 (23.8) | 93 (29.2) | 1.45 (0.15) | 0.066 |
| Blood Pressure (mm Hg) |  |  | 0.38 (0.54) | 0.113 |
| *≤130/90* | 60 (63.8) | 65 (69.1) |  |  |
| *>130/90* | 34 (36.2) | 29 (30.9) |  |  |
| **Comorbidities** |  |  |  |  |
| Any comorbidity | 46 (48.9) | 47 (50.0) | <0.01 (>0.99) | 0.021 |
| Tuberculosis | 0 (0.00) | 0 (0.0) | NA | NA |
| Heart disease (CVD) | 17 (18.1) | 15 (16.0) | 0.04 (0.85) | 0.057 |
| Asthma | 3 (3.19) | 2 (2.13) | <0.01 (>0.99) | 0.066 |
| COPD | 2 (2.13) | 0 (0.00) | 0.51 (0.48) | 0.209 |
| Diabetes | 25 (26.6) | 29 (30.9) | 0.23 (0.63) | 0.094 |
| Cancer | 1 (1.06) | 1 (1.06) | <0.01 (>0.99) | <0.001 |
| HIV | 9 (9.57) | 12 (12.8) | 0.21 (0.64) | 0.101 |
| **Oxygen therapy at admission** |  |  | 3.49 (0.18) | 0.274 |
| *No Supplemental O2* | 57 (60.6) | 69 (73.4) |  |  |
| *<10 l/min* | 23 (24.5) | 16 (17.0) |  |  |
| *10+ l/min* | 14 (14.9) | 9 (9.57) |  |  |
| **Vaccination status** |  |  | 0.22 (0.90) | 0.023 |
| *At least 1 dose of COVID-19 vaccine* | 29 (30.9) | 30 (31.9) |  |  |
| *1 dose* | 10 (10.6) | 12 (12.8) |  |  |
| *2 doses* | 19 (20.2) | 18 (19.1) |  |  |
| *Not vaccinated* | 65 (69.1) | 64 (68.1) |  |  |
| **Co-prescribed COVID-19 medications** |  |  |  |  |
| Dexamethasone | 70 (74.5) | 68 (72.3) | 0.03 (0.87) | 0.048 |
| Inhaled budesonide | 7 (7.45) | 7 (7.45) | <0.01 (>0.99) | <0.001 |
| Antibiotics | 48 (51.1) | 46 (48.9) | 0.02 (0.88) | 0.043 |
| **COVID-19 diagnostic methods** |  |  | <0.01 (>0.99) | 0.085 |
| *Positive RT-PCR* | 2 (2.13) | 1 (1.06) |  |  |
| *Positive RDT* | 92 (97.9) | 93 (98.9) |  |  |

^a^ We used Chi-squared test (χ2) or Fisher’s exact tests if necessary for categorical variables and two-sample Mood’s median tests for continuous variables.

**eTable 3. Clinical characteristics of participants treated and not treated with fluvoxamine in the 1:1 ratio matched analytic sample of patients who survived (N=130).**

|  | **Fluvoxamine**  **N=65** | **No fluvoxamine**  **N=65** | **Fluvoxamine vs. No fluvoxamine** | |
| --- | --- | --- | --- | --- |
|  | **N (%)** | **N (%)** | **χ^2^ (p-value) /**  **Z (p-value) ^a^** | **SMD** |
| **Characteristics** |  |  |  |  |
| Age (years) - *Median (IQR)* | 55.1 (20.9) | 18.0 (34.0) | -1.22 (0.22) | 0.266 |
| Sex |  |  | <0.01 (>0.99) | <0.001 |
| *Male* | 30 (46.2) | 30 (46.2) |  |  |
| *Female* | 35 (53.8) | 35 (53.8) |  |  |
| **COVID-19 symptoms** |  |  |  |  |
| Any symptom | 64 (98.5) | 63 (96.9) | <0.01 (>0.99) | 0.103 |
| Fever | 24 (36.9) | 17 (26.2) | 1.28 (0.26) | 0.233 |
| Cough | 55 (84.6) | 48 (73.8) | 1.68 (0.19) | 0.268 |
| Dyspnea | 27 (41.5) | 27 (41.5) | <0.01 (>0.99) | <0.001 |
| Muscle ache | 6 (9.23) | 6 (9.23) | <0.01 (>0.99) | <0.001 |
| Delirium | 0 (0.00) | 0 (0.0) | NA | NA |
| Headache | 13 (20.0) | 13 (20.0) | <0.01 (>0.99) | <0.001 |
| Pharyngitis | 0 (0.00) | 0 (0.0) | NA | NA |
| Rhinorrhea | 1 (1.54) | 1 (1.54) | <0.01 (>0.99) | <0.001 |
| Chest pain | 26 (40.0) | 17 (26.2) | 2.22 (0.14) | 0.298 |
| Diarrhea | 2 (3.08) | 2 (3.08) | <0.01 (>0.99) | <0.001 |
| Nausea or vomiting | 1 (1.54) | 2 (3.08) | <0.01 (>0.99) | 0.103 |
| **Vital signs** |  |  |  |  |
| Temperature (°C) - *Median (IQR)* | 37.1 (1.16) | 36.6 (2.10) | -1.57 (0.12) | 0.314 |
| Respiratory rate (breaths *per* minute) - *Median (IQR)* | 15 (31.5) | 15 (31.0) | 0.35 (0.73) | 0.086 |
| Pulse rate (bpm) - *Median (IQR)* | 91 (20.0) | 32 (24.0) | 1.22 (0.22) | 0.297 |
| Blood Pressure (mm Hg) |  |  | <0.01 (>0.99) | 0.031 |
| *≤130/90* | 36 (55.4) | 35 (53.8) |  |  |
| *>130/90* | 29 (44.6) | 30 (46.2) |  |  |
| **Comorbidities** |  |  |  |  |
| Any comorbidity | 30 (46.2) | 28 (43.1) | 0.03 (0.86) | 0.062 |
| Tuberculosis | 0 (0.00) | 0 (0.0) | NA | NA |
| Heart disease (CVD) | 15 (23.1) | 14 (21.5) | <0.01 (>0.99) | 0.037 |
| Asthma | 3 (4.62) | 1 (1.54) | 0.26 (0.61) | 0.179 |
| COPD | 2 (3.08) | 0 (0.00) | 0.51 (0.48) | 0.252 |
| Diabetes | 16 (24.6) | 18 (27.7) | 0.04 (0.84) | 0.070 |
| Cancer | 1 (1.54) | 3 (4.62) | 0.26 (0.61) | 0.179 |
| HIV | 2 (3.08) | 5 (7.69) | 0.60 (0.44) | 0.206 |
| **Oxygen therapy at admission** |  |  | 5.54 (0.07) | 0.412 |
| *No Supplemental O2* | 30 (46.2) | 43 (66.2) |  |  |
| *<10 l/min* | 22 (33.8) | 13 (20.0) |  |  |
| *10+ l/min* | 13 (20.0) | 9 (13.8) |  |  |
| **Vaccination status** |  |  | 0.18 (0.91) | 0.064 |
| *At least 1 dose of COVID-19 vaccine* | 22 (33.8) | 24 (36.9) |  |  |
| *1 dose* | 8 (12.3) | 8 (12.3) |  |  |
| *2 doses* | 14 (21.5) | 16 (24.6) |  |  |
| *Not vaccinated* | 43 (66.2) | 41 (63.1) |  |  |
| **Co-prescribed COVID-19 medications** |  |  |  |  |
| Dexamethasone | 46 (70.8) | 40 (61.5) | 0.86 (0.35) | 0.196 |
| Inhaled budesonide | 2 (3.08) | 2 (3.08) | <0.01 (>0.99) | <0.001 |
| Antibiotics | 42 (64.6) | 43 (66.2) | <0.01 (>0.99) | 0.032 |
| **COVID-19 diagnostic methods** |  |  | 0.51 (0.48) | 0.252 |
| *Positive RT-PCR* | 2 (3.08) | 0 (0.00) |  |  |
| *Positive RDT* | 63 (96.9) | 65 (100) |  |  |

^a^ We used Chi-squared test (χ2) or Fisher’s exact test if necessary for categorical variables and Asymptotic Two-Sample Brown-Mood Median test for continuous variables.

**eTable 4. Association of fluvoxamine with all-cause mortality and complete symptom resolution, and, among survivors, with hospital discharge in the matched analytic samples.**

|  | **Patients with fluvoxamine** | **Patients without fluvoxamine** | **Univariate Cox regression in the matched analytic sample ^a^** | **Multivariate Cox regression in the matched analytic sample adjusted for unbalanced covariates** |
| --- | --- | --- | --- | --- |
|  | **Events / N (%)** | **Events / N (%)** | **HR (95%CI; p-value)** | **AHR (95%CI; p-value)** |
| **Mortality** |  |  |  |  |
| *Fluvoxamine* | 29 / 94 (30.9) | 53 / 94 (56.4) | 0.38 (0.24 – 0.61; <0.001) | 0.44 (0.28-0.70; 0.001) ^b^ |
| **Hospital discharge among survivors** |  |  |  |  |
| *Fluvoxamine* | 65 / 65 (100) | 65 / 65 (100) | 0.75 (0.53 – 1.07; 0.11) | 0.88 (0.58-1.33; 0.54) ^c^ |
|  | **Patients with fluvoxamine** | **Patients without fluvoxamine** | **Univariate logistic regression in the matched analytic sample** | **Multivariate logistic regression in the matched analytic sample adjusted for unbalanced covariates** |
|  | **Events / N (%)** | **Events / N (%)** | **OR (95%CI; p-value)** | **AOR (95%CI; p-value)** |
| **Symptom resolution among survivors** |  |  |  |  |
| *Fluvoxamine* | 51 / 94 (54.3) | 31 / 94 (33.0) | 2.41 (1.34-4.39; 0.004) | 2.89 (2.97-4.83; 0.01) ^b^ |

^a^ The matched analytic sample was performed based on age, sex, fever, cough, dyspnea, muscle ache, delirium, headache, pharyngitis, rhinorrhea, chest pain, diarrhea, and nausea or vomiting, temperature, respiratory rate, pulse rate, blood pressure, tuberculosis, heart disease, asthma, COPD, diabetes, cancer, HIV, oxygen therapy at admission, vaccination status, dexamethasone, inhaled budesonide, antibiotics, and method of COVID-19 diagnosis (degrees of freedom=31). The exposed/non-exposed ratio was *a priori* defined as 1:1 in the full sample and as 1:1 among survivors.

^b^ Adjusted for fever, cough, headache, diarrhea, respiratory rate, blood pressure, COPD, HIV, and oxygen therapy at admission.

^c^ Adjusted for age, fever, cough, chest pain, nausea or vomiting, temperature, pulse rate, asthma, COPD, cancer, HIV, oxygen therapy at admission, dexamethasone, and method of COVID-19 diagnosis.

**eTable 5. Association of fluvoxamine with all-cause mortality at Day 15 and Day 28.**

|  | **Patients with fluvoxamine** | **Patients without fluvoxamine** | **Crude Cox regression analysis** | **Multivariable Cox regression analysis ^a^** | **Cox regression analysis weighted by IPW** | **Cox regression analysis weighted by IPW adjusted for unbalanced covariates** | **Cox regression analysis weighted by IPW adjusted for unbalanced covariates after excluding outliers** |
| --- | --- | --- | --- | --- | --- | --- | --- |
|  | **Events / N (%)** | **Events / N (%)** | **HR (95%CI; p-value)** | **AHR (95%CI; p-value)** | **HR (95%CI; p-value)** | **AHR (95%CI; p-value)** | **AHR (95%CI; p-value) [number of outliers]** |
| **Censoring at Day 15** |  |  |  |  |  |  |  |
| Mortality |  |  |  |  |  |  |  |
| *Fluvoxamine* | 28 / 94 (29.8) | 125 / 222 (56.3) | 0.43 (0.21 - 0.66; <0.001) | 0.47 (0.30-0.75; 0.001) | 0.32 (0.14 - 0.71; 0.005) | 0.23 (0.13-0.41; <0.001) ^b^ | 0.20 (0.11-0.35; <0.001) [8] |
| **Censoring at Day 28** |  |  |  |  |  |  |  |
| Mortality |  |  |  |  |  |  |  |
| *Fluvoxamine* | 29 / 94 (30.9) | 125 / 222 (56.3) | 0.44 (0.30 - 0.67; <0.001) | 0.48 (0.31-0.76; 0.002) | 0.33 (0.15 - 0.72; 0.005) | 0.24 (0.14-0.42; <0.001)  ^b^ | 0.20 (0.11-0.35; <0.001) [9] |

^a^ Adjusted for age, sex, fever, cough, dyspnea, muscle ache, delirium, headache, pharyngitis, rhinorrhea, chest pain, diarrhea, and nausea or vomiting, temperature, respiratory rate, pulse rate, blood pressure, tuberculosis, heart disease, asthma, COPD, diabetes, cancer, HIV, oxygen therapy at admission, vaccination status, dexamethasone, inhaled budesonide, antibiotics, and method of COVID-19 diagnosis (degrees of freedom=31, all GVIFs <2.5).

^b^ Adjusted for temperature, respiratory rate, tuberculosis, cancer and antibiotics.

**eTable 6. Association of fluvoxamine with all-cause mortality, by clinical characteristic.**

|  | **Patients with fluvoxamine** | **Patients without fluvoxamine** | **Crude Cox regression analysis** | **Multivariable Cox regression analysis ^a^** | **Cox regression analysis weighted by IPW** | **Cox regression analysis weighted by IPW adjusted for unbalanced covariates** | **Cox regression model testing the interaction characteristic*fluvoxamine while including the characteristic and fluvoxamine** |
| --- | --- | --- | --- | --- | --- | --- | --- |
|  | **Events / N (%)** | **Events / N (%)** | **HR (95%CI; p-value)** | **AHR (95%CI; p-value)** | **HR (95%CI; p-value)** | **AHR (95%CI; p-value)** | **IHR (95%CI; p-value)** |
| **Full sample (N=316)** |  |  |  |  |  |  |  |
| *Fluvoxamine* | 29 / 94 (30.9) | 126 / 222 (56.8) | 0.44 (0.30-0.37; <0.001) | 0.29 (0.18-0.47; <0.001) | 0.33 (0.15-0.72; 0.005) | 0.32 (0.19-0.53; <0.001)^b^ | NA |
| **Age ≤ 60 (N=162)** |  |  |  |  |  |  |  |
| *Fluvoxamine* | 12 / 47 (25.5) | 47 / 114 (41.2) | 0.56 (0.30-1.07; 0.08) | 0.30 (0.14-0.67; <0.001) | 0.86 (0.41-1.82; 0.69) | 0.45 (0.23-0.91; 0.03) ^c^ | Ref. |
| **Age > 60 (N=154)** |  |  |  |  |  |  |  |
| *Fluvoxamine* | 17 / 47 (25.5) | 79 / 108 (73.1) | 0.35 (0.20-0.59; <0.001) | 0.39 (0.19-0.78; <0.001) | 0.24 (0.07-0.78; 0.02) | 0.23 (0.11-0.49; <0.001) ^d^ | 1.95 (0.08-1.10; 0.07) |
| **Male (N=151)** |  |  |  |  |  |  |  |
| *Fluvoxamine* | 15 / 45 (33.3) | 57 / 106 (53.8) | 0.49 (0.28-0.87; 0.02) | 0.28 (0.14-0.57; <0.001) | 0.88 (0.48-1.61; 0.68) | 0.62 (0.37-1.04; 0.07) ^e^ | Ref |
| **Female (N=165)** |  |  |  |  |  |  |  |
| *Fluvoxamine* | 14 / 49 (28.6) | 69 / 116 (59.5) | 0.38 (0.21-0.69; 0.001) | 0.35 (0.16-0.78; <0.001) | 0.66 (0.29-1.48; 0.31) | 0.10 (0.04-0.23; <0.001) ^f^ | 0.28 (0.07-1.06; 0.06) |
| **With any symptom (N=297)** |  |  |  |  |  |  |  |
| *Fluvoxamine* | 28 / 92 (30.4) | 119 / 205 (58.0) | 0.42 (0.28-0.64; <0.001) | 0.33 (0.21-0.54; <0.001) | 0.31 (0.14-0.69; 0.004) | 0.29 (0.15-0.54; <0.001) ^g^ | 0.09 (0.01-1.24; 0.07) |
| **No symptoms (N=19)** |  |  |  |  |  |  |  |
| *Fluvoxamine* | 1 / 2 (50.0) | 7 / 17 (43.8) | NA | NA | NA | NA | Ref. |
| **Temperature ≤37.2ºC (N=162)** |  |  |  |  |  |  |  |
| *Fluvoxamine* | 8 / 41 (19.5) | 57 / 121 (47.1) | 0.38 (0.18-0.80; 0.01) | 0.18 (0.07-0.46; <0.001) | NP | NP | Ref. |
| **Temperature >37.2ºC (N=154)** |  |  |  |  |  |  |  |
| *Fluvoxamine* | 21 / 53 (39.6) | 69 / 101 (68.3) | 0.40 (0.25-0.66; <0.001) | 0.68 (0.38-1.23; 0.20) | 0.46 (0.28-0.76; 0.003) | 0.46 (0.29-0.71; <0.001) ^e^ | NA |
| **Respiratory rate ≤20 (N=171)** |  |  |  |  |  |  |  |
| *Fluvoxamine* | 12 / 56 (21.4) | 54 / 117 (46.2) | 0.41 (0.22-0.77; 0.005) | 0.38 (0.15-0.97; 0.04) | 0.33 (0.15-0.70; 0.004) | 0.54 (0.22-1.31; 0.17) ^h^ | Ref. |
| **Respiratory rate**  **>20 (N=145)** |  |  |  |  |  |  |  |
| *Fluvoxamine* | 17 / 38 (44.7) | 72 / 105 (68.6) | 0.51 (0.30-0.87; 0.013) | 0.42 (0.23-0.76; <0.001) | 0.40 (0.22-0.75; 0.004) | 0.59 (0.36-0.96; 0.03) ^i^ | 0.63 (0.16-2.49; 0.51) |
| **Pulse rate ≤92 (N=139)** |  |  |  |  |  |  |  |
| *Fluvoxamine* | 12 / 57 (21.1) | 38 / 81 (46.9) | 0.40 (0.21-0.76; 0.005) | 0.30 (0.12-0.74; <0.001) | 0.21 (0.10-0.48; <0.001) | 0.28 (0.12-0.62; <0.001) ^j^ | Ref. |
| **Pulse rate >92 (N=178)** |  |  |  |  |  |  |  |
| *Fluvoxamine* | 17 / 37 (45.9) | 88 / 141 (62.4) | 0.57 (0.34-0.96; 0.03) | 0.56 (0.33-0.96; 0.04) | 0.58 (0.29-1.19; 0.14) | 0.35 (0.19-0.63; <0.001) ^k^ | 0.90 (0.23-3.46; 0.88) |
| **Blood pressure ≤130/90 mm Hg (N=218)** |  |  |  |  |  |  |  |
| *Fluvoxamine* | 24 / 60 (40.0) | 103 / 158 (65.2) | 0.50 (0.32-0.79; 0.003) | 0.30 (0.18-0.51; <0.001) | 0.26 (0.10-0.73; 0.01) | 0.28 (0.17-0.45; <0.001) ^l^ | Ref. |
| **Blood pressure >130/90 mm Hg (N=98)** |  |  |  |  |  |  |  |
| *Fluvoxamine* | 5 / 34 (14.7) | 23 / 64 (35.9) | 0.35 (0.13-0.93; 0.03) | 0.35 (0.13-0.93; 0.03) | 0.31 (0.12-0.79; 0.01) | 0.28 (0.17-0.45; <0.001) ^l^ | 1.37 (0.29-6.61; 0.69) |
| **Any comorbidity (N=153)** |  |  |  |  |  |  |  |
| *Fluvoxamine* | 16 / 46 (34.8) | 58 / 107 (54.2) | 0.54 (0.31-0.95; 0.03) | 0.12 (0.05-0.28; <0.001) | 0.45 (0.25-0.80; 0.007) | 0.50 (0.27-0.92; 0.03) ^m^ | 1.97 (0.53-7.32; 0.31) |
| **No comorbidities (N=163)** |  |  |  |  |  |  |  |
| *Fluvoxamine* | 13 / 48 (27.0) | 68 / 115 (59.1) | 0.38 (0.21-0.69; 0.01) | 0.38 (0.18-0.79; <0.001) | 0.29 (0.11-0.78; 0.01) | 0.24 (0.12-0.48; <0.001) ^n^ | Ref. |
| **Tuberculosis (N=11)** |  |  |  |  |  |  |  |
| *Fluvoxamine* | 0 / 0 (0.0) | 7 / 11 (63.6) | NA | NA | NA | NA | NA |
| **No tuberculosis (N=305)** |  |  |  |  |  |  |  |
| *Fluvoxamine* | 29 / 94 (30.9) | 119 / 211 (56.4) | 0.43 (0.28-0.64; <0.001) | 0.38 (0.24-0.6; <0.001) | 0.32 (0.14-0.69; 0.004) | 0.30 (0.19-0.50; <0.001) ^o^ | Ref. |
| **Heart disease (N=49)** |  |  |  |  |  |  |  |
| *Fluvoxamine* | 2 / 32 (6.3) | 10 / 17 (58.8) | NA | NA | NA | NA | NA |
| **No heart disease (N=267)** |  |  |  |  |  |  |  |
| *Fluvoxamine* | 27 / 77 (35.1) | 116 / 190 (61.1) | 0.46 (0.30-0.70; <0.001) | 0.39 (0.24-0.63; <0.001) | 0.31 (0.12-0.76; 0.01) | 0.31 (0.19-0.50; <0.001) ^o^ | Ref. |
| **Asthma (N=8)** |  |  |  |  |  |  |  |
| *Fluvoxamine* | 0 / 3 (0.0) | 4 / 5 (80.0) | NA | NA | NA | NA | NA |
| **No asthma (N=308)** |  |  |  |  |  |  |  |
| *Fluvoxamine* | 29 / 91 (31.9) | 122 / 217 (56.2) | 0.50 (0.31-0.70; <0.001) | 0.39 (0.24-0.61; <0.001) | 0.34 (0.16-0.73; 0.005) | 0.32 (0.2-0.52; <0.001) ^o^ | Ref. |
| **COPD (N=3)** |  |  |  |  |  |  |  |
| *Fluvoxamine* | 0 / 2 (0.0) | 1 / 1 (100) | NA | NA | NA | NA | NA |
| **No COPD (N=313)** |  |  |  |  |  |  |  |
| *Fluvoxamine* | 29 / 92 (31.5) | 125 / 221 (56.6) | 0.46 (0.30-0.68; <0.001) | 0.37 (0.23-0.59; <0.001) | 0.33 (0.15-0.72; 0.006) | 0.31 (0.19-0.51; <0.001) ^o^ | Ref. |
| **Diabetes (N=83)** |  |  |  |  |  |  |  |
| *Fluvoxamine* | 9 / 25 (36.0) | 33 / 58 (56.9) | 0.51 (0.24-1.07; 0.07) | NA | NA | NA | 0.92 (0.25-3.32; 0.89) |
| **No diabetes (N=233)** |  |  |  |  |  |  |  |
| *Fluvoxamine* | 20 / 69 (29.0) | 93 / 164 (56.7) | 0.43 (0.27-0.70; 0.001) | 0.40 (0.23-0.71; <0.001) | 0.29 (0.12-0.72; 0.007) | 0.20 (0.11-0.37; <0.001) ^p^ | Ref. |
| **Cancer (N=8)** |  |  |  |  |  |  |  |
| *Fluvoxamine* | 0 / 1 (0.0) | 2 / 7 (28.6) | NA | NA | NA | NA | NA |
| **No cancer (N=308)** |  |  |  |  |  |  |  |
| *Fluvoxamine* | 29 / 93 (31.2) | 124 / 215 (57.7) | 0.44 (0.29-0.65; <0.001) | 0.37 (0.23-0.58; <0.001) | 0.33 (0.151-0.73; 0.006) | 0.32 (0.2-0.54; <0.001) ^o^ | Ref. |
| **HIV (N=42)** |  |  |  |  |  |  |  |
| *Fluvoxamine* | 7 / 9 (77.8) | 17 / 33 (51.5) | 1.41 (0.57-3.45; 0.45) | 0.40 (0.14-1.11; 0.08) | 1.41 (0.65-3.03; 0.38) | 0.35 (0.10-1.19; 0.09) ^q^ | 3.34 (0.86-12.97; 0.08) |
| **No HIV (N=274)** |  |  |  |  |  |  |  |
| *Fluvoxamine* | 22 / 85 (25.9) | 109 / 189 (57.8) | 0.36 (0.23-0.57; <0.001) | 0.36 (0.21-0.62; <0.001) | 0.24 (0.09-0.61; 0.003) | 0.17 (0.1-0.28; <0.001) ^r^ | Ref. |
| **No Supplemental Oxygen (N=237)** |  |  |  |  |  |  |  |
| *Fluvoxamine* | 27 / 57 (47.4) | 116 / 180 (64.4) | 0.74 (0.49-1.13; 0.16) | 0.43 (0.27-0.69; <0.001) | 0.30 (0.10-0.93; 0.04) | 0.31 (0.17-0.56; <0.001) ^s^ | Ref. |
| **Supplemental Oxygen (N=79)** |  |  |  |  |  |  |  |
| *Fluvoxamine* | 2 / 37 (5.4) | 10/ 42 (23.8) | 0.17 (0.04-0.80; 0.03) | NA | NA | NA | 0.23 (0.05-1.11; 0.07) |
| **<10 L/min (N=50)** |  |  |  |  |  |  |  |
| *Fluvoxamine* | 1 / 23 (4.3) | 6 / 27 (22.2) | 0.16 (0.02-1.34; 0.09) | NA | NA | NA | 0.36 (0.03-3.63; 0.35) |
| **≥10 L/min (N=29)** |  |  |  |  |  |  |  |
| *Fluvoxamine* | 1 / 14 (7.1) | 4 / 15 (26.7) | 0.21 (0.02-1.89; 0.16) | NA | NA | NA | 0.35 (0.03-4.03; 0.40) |
| **With vaccine – 1 dose (N=33)** |  |  |  |  |  |  |  |
| *Fluvoxamine* | 2 / 10 (20.0) | 12 / 23 (52.2) | NA | NA | NA | NA | 0.86 (0.20-3.69; 0.840) |
| **With vaccine – 2 doses (N=48)** |  |  |  |  |  |  |  |
| *Fluvoxamine* | 5 / 19 (26.3) | 11 / 29 (37.9) | 0.61 (0.21-1.77; 0.360) | NA | NA | NA | 1.89 (0.38-9.34; 0.433) |
| **With vaccine – At least 1 dose (N=81)** |  |  |  |  |  |  |  |
| *Fluvoxamine* | 7 / 29 (24.1) | 23 / 52 (44.2) | 0.39 (0.16-0.95; 0.038) | NA | NA | NA | 1.41 (0.36-5.49; 0.617) |
| **Not vaccinated (N=235)** |  |  |  |  |  |  |  |
| *Fluvoxamine* | 22 / 65 (33.8) | 103 / 170 (60.6) | 0.46 (0.29-0.73; 0.001) | 0.36 (0.21-0.61; <0.001) | 0.27 (0.09-0.75; 0.012) | 0.28 (0.18-0.45; <0.001) ^t^ | Ref. |
| **Dexamethasone (N=232)** |  |  |  |  |  |  |  |
| *Fluvoxamine* | 24 / 70 (34.3) | 106 / 162 (65.4) | 0.40 (0.25-0.62; <0.001) | 0.36 (0.21-0.63; <0.001) | 0.35 (0.18-0.72; 0.004) | 0.40 (0.20-0.78; <0.001) ^e^ | 0.46 (0.10-2.18; 0.329) |
| **No dexamethasone (N=84)** |  |  |  |  |  |  |  |
| *Fluvoxamine* | 5 / 24 (20.8) | 20 / 60 (33.3) | 0.58 (0.22-1.55; 0.276) | 0.24 (0.08-0.7; <0.001) | 0.25 (0.06-1.00; 0.051) | 0.13 (0.03-0.57; <0.001) ^u^ | Ref. |
| **Inhaled budesonide (N=16)** |  |  |  |  |  |  |  |
| *Fluvoxamine* | 5 / 7 (71.4) | 6 / 9 (66.7) | 0.83 (0.23-2.96; 0.776) | NA | NA | NA | 1.15 (0.24-5.54; 0.859) |
| **No inhaled budesonide (N=300)** |  |  |  |  |  |  |  |
| *Fluvoxamine* | 24 / 87 (27.6) | 120 / 213 (56.3) | 0.41 (0.26-0.64; <0.001) | 0.39 (0.24-0.64; <0.001) | 0.31 (0.14-0.72; 0.006) | 0.32 (0.2-0.53; <0.001) ^o^ | Ref. |
| **Antibiotics (N=159)** |  |  |  |  |  |  |  |
| *Fluvoxamine* | 6 / 48 (12.5) | 46 / 111 (41.4) | 0.25 (0.11-0.59; 0.002) | 0.23 (0.07-0.80; 0.02) | 0.38 (0.14-1.05; 0.06) | 0.45 (0.14-1.44; 0.18) ^v^ | 1.37 (0.33-5.72; 0.666) |
| **No antibiotics (N=157)** |  |  |  |  |  |  |  |
| *Fluvoxamine* | 23 / 46 (50.0) | 80 / 111 (72.1) | 0.55 (0.35-0.88; 0.013) | 0.47 (0.27-0.81; <0.001) | 0.23 (0.08-0.67; 0.007) | 0.25 (0.15-0.41; <0.001) ^w^ | Ref. |
| **Positive RT-PCR (N=5)** |  |  |  |  |  |  |  |
| *Fluvoxamine* | 0 / 2 (0.0) | 1 / 3 (33.3) | NA | NA | NA | NA | NA |
| **Positive RDT (N=311)** |  |  |  |  |  |  |  |
| *Fluvoxamine* | 29 / 92 (31.5) | 125 / 219 (57.1) | 0.46 (0.30-0.68; <0.001) | 0.38 (0.24-0.6; <0.001) | 0.34 (0.16-0.74; 0.007) | 0.33 (0.20-0.55; <0.001) ^o^ | Ref. |

^a^ Adjusted for age, sex, fever, cough, dyspnea, muscle ache, delirium, headache, pharyngitis, rhinorrhea, chest pain, diarrhea, and nausea or vomiting, temperature, respiratory rate, pulse rate, blood pressure, tuberculosis, heart disease, asthma, COPD, diabetes, cancer, HIV, oxygen therapy at admission, vaccination status, dexamethasone, inhaled budesonide, antibiotics, and method of COVID-19 diagnosis (degrees of freedom=31, all GVIFs <1.9).

^b^ Adjusted for temperature, respiratory rate, tuberculosis, cancer, and antibiotics.

^c^ Adjusted for dyspnea.

^d^ Adjusted for age, sex, fever, cough, dyspnea, chest pain, respiratory rate, pulse rate, blood pressure, diabetes, vaccination status and antibiotics.

^e^ Adjusted for age and pulse rate.

^f^ Adjusted for age, fever, cough, dyspnea, headache, chest pain, temperature, respiratory rate, pulse rate, blood pressure, diabetes, oxygen therapy at admission, vaccination status, dexamethasone and antibiotics.

^g^ Adjusted for age, respiratory rate and pulse rate.

^h^ Adjusted for age, pulse rate and heart disease.

^i^ Adjusted for sex and temperature.

^j^ Adjusted for age, respiratory rate, diabetes and oxygen therapy at admission.

^k^ Adjusted for age, respiratory rate, blood pressure, diabetes, vaccination status and antibiotics.

^l^ Adjusted for age, sex, cough, dyspnea, temperature, respiratory rate, pulse rate and antibiotics.

^m^ Adjusted for fever, cough, dyspnea, temperature and blood pressure.

^n^ Adjusted for age, cough, temperature, respiratory rate, pulse rate, blood pressure and antibiotics.

^o^ Adjusted for age, cough, respiratory rate, pulse rate and antibiotics.

^p^ Adjusted for age, sex, fever, cough, dyspnea, chest pain, temperature, respiratory rate, pulse rate, vaccination status and antibiotics.

^q^ Adjusted for age, fever, cough, dyspnea, muscle ache, headache, temperature, respiratory rate, tuberculosis, diabetes, vaccination status and antibiotics.

^r^ Adjusted for age, sex, cough, temperature, respiratory rate, pulse rate, blood pressure, vaccination status and antibiotics.

^s^ Adjusted for age, fever, cough, dyspnea, temperature, respiratory rate, pulse rate, diabetes and antibiotics.

^t^ Adjusted for age, cough, dyspnea, chest pain, respiratory rate, pulse rate, blood pressure, diabetes and antibiotics.

^u^ Adjusted for age, sex, fever, dyspnea, headache, chest pain, respiratory rate, blood pressure, HIV and antibiotics.

^v^ Adjusted for age, respiratory rate, pulse rate and heart disease.

^w^ Adjusted for age, cough, dyspnea, temperature, respiratory rate, pulse rate and diabetes.

Abbreviations: IPW, inverse-probability-weighting weights; HR, hazard ratio; AHR, adjusted hazard ratio; IHR, hazard ratio for the interaction term; NP, not performed because no covariate substantially differed between groups in the crude analysis (all SMDs <0.1); NA, not applicable.

**eTable 7. Association of fluvoxamine with complete symptom resolution, by clinical characteristic.**

|  | **Patients with fluvoxamine** | **Patients without fluvoxamine** | **Crude logistic regression analysis** | **Multivariable logistic regression analysis ^a^** | **Cox regression model testing the interaction characteristic*fluvoxamine while including the characteristic and fluvoxamine** |
| --- | --- | --- | --- | --- | --- |
|  | **Events / N (%)** | **Events / N (%)** | **OR (95%CI; p-value)** | **AOR (95%CI; p-value)** | **IOR (95%CI; p-value)** |
| **Full sample (N=316)** |  |  |  |  |  |
| *Fluvoxamine* | 51 / 94 (54.3) | 69 / 222 (31.1) | 2.63 (1.61-4.33; <0.001) | 2.56 (1.16-5.78; 0.008) | NA |
| **Age ≤ 60 (N=161)** |  |  |  |  |  |
| *Fluvoxamine* | 25 / 47 (53.2) | 49 / 114 (43) | 1.51 (0.76-3; 0.24) | 1.62 (0.54-5.02; 0.40) | Ref. |
| **Age > 60 (N=155)** |  |  |  |  |  |
| *Fluvoxamine* | 26 / 47 (55.3) | 20 / 108 (18.5) | 5.45 (2.59-11.75; <0.001) | 5.50 (2.14-14.97; <0.001) | 2.26 (0.51-10.09; 0.28) |
| **Male (N=151)** |  |  |  |  |  |
| *Fluvoxamine* | 24 / 45 (53.3) | 36 / 106 (34) | 2.22 (1.1-4.56; 0.03) | 2.08 (0.55-8.11; 0.28) | Ref. |
| **Female (N=165)** |  |  |  |  |  |
| *Fluvoxamine* | 27 / 49 (55.1) | 33 / 116 (28.4) | 3.09 (1.55-6.23; 0.001) | 3.64 (1.48-9.25; 0.005) | 1.20 (0.27-5.41; 0.81) |
| **With any symptom (N=297)** |  |  |  |  |  |
| *Fluvoxamine* | 50 / 92 (54.3) | 62 / 205 (30.2) | 2.75 (1.66-4.58; <0.001) | 2.52 (1.11-5.87; 0.03) | 0.35 (0.01-13.58; 0.54) |
| **No symptoms (N=19)** |  |  |  |  |  |
| *Fluvoxamine* | 1 / 2 (50) | 7 / 17 (41.2) | NA | NA | Ref. |
| **Temperature ≤37.2ºC (N=162)** |  |  |  |  |  |
| *Fluvoxamine* | 26 / 41 (63.4) | 49 / 121 (40.5) | 2.55 (1.24-5.39; 0.01) | 3.24 (1.19-9.26; 0.02) | Ref. |
| **Temperature >37.2ºC (N=154)** |  |  |  |  |  |
| *Fluvoxamine* | 25 / 53 (47.2) | 20 / 101 (19.8) | 3.62 (1.76-7.58; <0.001) | 3.75 (1.54-9.54; 0.004) | 1.71 (0.38-7.79; 0.48) |
| **Respiratory rate ≤20 (N=173)** |  |  |  |  |  |
| *Fluvoxamine* | 42 / 56 (75) | 60 / 117 (51.3) | 2.85 (1.43-5.92; 0.004) | 2.83 (1.14-7.4; 0.03) | Ref. |
| **Respiratory rate**  **>20 (N=143)** |  |  |  |  |  |
| *Fluvoxamine* | 9 / 38 (23.7) | 9 / 105 (8.6) | 3.31 (1.19-9.26; 0.02) | NA | 1.66 (0.29-9.68; 0.57) |
| **Pulse rate ≤92 (N=138)** |  |  |  |  |  |
| *Fluvoxamine* | 35 / 57 (61.4) | 30 / 81 (37) | 2.7 (1.36-5.5; 0.005) | 2.96 (1.27-7.15; 0.01) | Ref. |
| **Pulse rate >92 (N=178)** |  |  |  |  |  |
| *Fluvoxamine* | 16 / 37 (43.2) | 39 / 141 (27.7) | 1.99 (0.93-4.21; 0.07) | 0.69 (0.11-3.91; 0.68) | 0.19 (0.03-1.09; 0.07) |
| **Blood pressure ≤130/90 mm Hg (N=218)** |  |  |  |  |  |
| *Fluvoxamine* | 25 / 60 (41.7) | 37 / 158 (23.4) | 2.34 (1.24-4.4; 0.008) | 2.47 (0.96-6.47; 0.06) | Ref. |
| **Blood pressure >130/90 mm Hg (N=98)** |  |  |  |  |  |
| *Fluvoxamine* | 26 / 34 (76.5) | 32 / 64 (50) | 3.25 (1.32-8.67; 0.01) | NA | 3.2 (0.55-20.96; 0.21) |
| **Any comorbidity (N=153)** |  |  |  |  |  |
| *Fluvoxamine* | 29 / 46 (63) | 38 / 107 (35.5) | 3.10 (1.53-6.45; 0.002) | 2.00 (0.84-4.80; 0.12) | 1.43 (0.48-4.22; 0.52) |
| **No comorbidities (N=163)** |  |  |  |  |  |
| *Fluvoxamine* | 22 / 48 (45.8) | 31 / 115 (27) | 2.29 (1.14-4.64; 0.02) | 1.39 (0.31-5.87; 0.66) | Ref. |
| **Tuberculosis (N=11)** |  |  |  |  |  |
| *Fluvoxamine* | 0 / 0 (0.0) | 0 / 11 (0.0) | NA | NA | NA |
| **No tuberculosis (N=305)** |  |  |  |  |  |
| *Fluvoxamine* | 51 / 94 (54.3) | 65 / 211 (30.8) | 2.66 (1.62-4.41; <0.001) | 2.09 (0.98-4.49; 0.06) | Ref. |
| **Heart disease (N=49)** |  |  |  |  |  |
| *Fluvoxamine* | 14 / 17 (82.4) | 21 / 32 (65.6) | 2.44 (0.63-12.25; 0.23) | NA | 3.67 (0.42-36.86; 0.25) |
| **No heart disease (N=267)** |  |  |  |  |  |
| *Fluvoxamine* | 37 / 77 (48.1) | 48 / 190 (25.3) | 2.74 (1.57-4.78; <0.001) | 1.48 (0.45-4.84; 0.51) | Ref. |
| **Asthma (N=8)** |  |  |  |  |  |
| *Fluvoxamine* | 3 / 3 (100) | 1 / 5 (20) | NA | NA | NA |
| **No asthma (N=308)** |  |  |  |  |  |
| *Fluvoxamine* | 48 / 91 (52.7) | 68 / 217 (31.3) | 2.45 (1.48-4.05; <0.001) | 2.09 (0.99-4.45; 0.05) | Ref. |
| **COPD (N=3)** |  |  |  |  |  |
| *Fluvoxamine* | 2 / 2 (100) | 0 / 1 (0) | NA | NA |  |
| **No COPD (N=313)** |  |  |  |  | NA |
| *Fluvoxamine* | 49 / 92 (53.3) | 69 / 221 (31.2) | 2.51 (1.53-4.15; <0.001) | 2.15 (1.02-4.59; 0.04) | Ref. |
| **Diabetes (N=83)** |  |  |  |  |  |
| *Fluvoxamine* | 16 / 25 (64) | 18 / 58 (31) | 3.95 (1.5-10.99; 0.006) | NA | 6.41 (0.9-51.12; 0.07) |
| **No diabetes (N=233)** |  |  |  |  |  |
| *Fluvoxamine* | 35 / 69 (50.7) | 51 / 164 (31.1) | 2.28 (1.28-4.07; 0.005) | 2.36 (1.13-4.99; 0.02) | Ref. |
| **Cancer (N=8)** |  |  |  |  |  |
| *Fluvoxamine* | 1 / 1 (100) | 4 / 7 (57.1) | NA | NA | NA |
| **No cancer (N=308)** |  |  |  |  |  |
| *Fluvoxamine* | 50 / 93 (53.8) | 65 / 215 (30.2) | 2.68 (1.63-4.45; <0.001) | 2.16 (1.01-4.67; 0.05) | Ref. |
| **HIV (N=42)** |  |  |  |  |  |
| *Fluvoxamine* | 2 / 9 (22.2) | 12 / 33 (36.4) |  |  | 0.07 (<0.01-1.16; 0.07) |
| **No HIV (N=274)** |  |  |  |  |  |
| *Fluvoxamine* | 49 / 85 (57.6) | 57 / 189 (30.2) | 3.15 (1.86-5.39; <0.001) | 2.27 (1.03-5.06; 0.04) | Ref. |
| **No Supplemental Oxygen (N=237)** |  |  |  |  |  |
| *Fluvoxamine* | 16 / 57 (28.1) | 39 / 180 (21.7) | 1.41 (0.7-2.75; 0.32) | 1.18 (0.49-2.77; 0.70) | Ref. |
| **Supplemental Oxygen (N=79)** |  |  |  |  |  |
| *Fluvoxamine* | 35 / 37 (94.6) | 30 / 42 (71.4) | 7.00 (1.73-47.32; 0.02) | NA | NA |
| **<10 L/min (N=50)** |  |  |  |  |  |
| *Fluvoxamine* | 22 / 23 (95.7) | 20 / 27 (74.1) | NA | NA | NA |
| **≥10 L/min (N=29)** |  |  |  |  |  |
| *Fluvoxamine* | 13 / 14 (92.9) | 10 / 15 (66.7) | NA | NA | NA |
| **With vaccine – 1 dose (N=33)** |  |  |  |  |  |
| *Fluvoxamine* | 6 / 10 (60) | 9 / 23 (39.1) | 2.33 (0.52-11.46; 0.27) | NA | 2.48 (0.19-37.61; 0.5) |
| **With vaccine – 2 doses (N=48)** |  |  |  |  |  |
| *Fluvoxamine* | 11 / 19 (57.9) | 15 / 29 (51.7) | 1.28 (0.40-4.21; 0.68) | NA | 0.70 (0.10-5.22; 0.73) |
| **With vaccine – At least 1 dose (N=81)** |  |  |  |  |  |
| *Fluvoxamine* | 17 / 29 (58.6) | 24 / 52 (46.2) | 1.65 (0.66-4.21; 0.28) | NA | 1.15 (0.2-6.86; 0.88) |
| **Not vaccinated (N=235)** |  |  |  |  |  |
| *Fluvoxamine* | 34 / 65 (52.3) | 45 / 170 (26.5) | 3.05 (1.69-5.55; <0.001) | 6.51 (1.28-38.0; 0.03) | Ref. |
| **Dexamethasone (N=232)** |  |  |  |  |  |
| *Fluvoxamine* | 38 / 70 (54.3) | 41 / 162 (25.3) | 3.5 (1.95-6.36; <0.001) | 3.11 (1.47-6.7; 0.003) | 2.59 (0.47-14.76; 0.28) |
| **No dexamethasone (N=84)** |  |  |  |  |  |
| *Fluvoxamine* | 13 / 24 (54.2) | 28 / 60 (46.7) | 1.35 (0.52-3.54; 0.54) | 1.47 (3.34-6.93; 0.608) | Ref. |
| **Inhaled budesonide (N=16)** |  |  |  |  |  |
| *Fluvoxamine* | 2 / 7 (28.6) | 2 / 9 (22.2) | NA | NA | 0.20 (0.01-5.71; 0.33) |
| **No inhaled budesonide (N=300)** |  |  |  |  |  |
| *Fluvoxamine* | 49 / 87 (56.3) | 67 / 213 (31.5) | 2.26 (1.05-4.95; 0.04) | 2.26 (1.05-4.95; 0.04) | Ref. |
| **Antibiotics (N=159)** |  |  |  |  |  |
| *Fluvoxamine* | 41 / 48 (85.4) | 62 / 111 (55.9) | 4.63 (2.01-12.08; <0.001) | 4.15 (1.11-17.9; 0.04) | 1.05 (0.16-6.86; 0.96) |
| **No antibiotics (N=157)** |  |  |  |  |  |
| *Fluvoxamine* | 10 / 46 (21.7) | 7 / 111 (6.3) | 4.13 (1.48-12.14; 0.007) | NA | Ref. |
| **Positive RT-PCR (N=5)** |  |  |  |  |  |
| *Fluvoxamine* | 2 / 2 (100) | 1 / 3 (33.3) | NA | NA | Ref. |
| **Positive RDT (N=311)** |  |  |  |  |  |
| *Fluvoxamine* | 49 / 92 (53.3) | 68 / 219 (31.1) | 2.53 (1.54-4.19; <0.001) | 3.46 (1.26-10.0; 0.02) | NA |

^a^ Adjusted for age, sex, fever, cough, dyspnea, muscle ache, delirium, headache, pharyngitis, rhinorrhea, chest pain, diarrhea, and nausea or vomiting, temperature, respiratory rate, pulse rate, blood pressure, tuberculosis, heart disease, asthma, COPD, diabetes, cancer, HIV, oxygen therapy at admission, vaccination status, dexamethasone, inhaled budesonide, antibiotics, and method of COVID-19 diagnosis (degrees of freedom=31, all GVIFs <1.9).

Abbreviations: OR, odds ratio; AOR, adjusted odds ratio; IOR, odds ratio for the interaction term; NP, not performed because no covariate substantially differed between groups in the crude analysis (all SMDs <0.1); NA, not applicable.
